# TRIB3 mediates vascular calcification by facilitating self-ubiquitination and dissociation of Smurf1 in chronic kidney disease

**DOI:** 10.1172/JCI175972

**Published:** 2025-04-01

**Authors:** Yihui Li, Chang Ma, Yanan Sheng, Shanying Huang, Huaibing Sun, Yun Ti, Zhihao Wang, Feng Wang, Fangfang Chen, Chen Li, Haipeng Guo, Mengxiong Tang, Fangqiang Song, Hao Wang, Ming Zhong

**Affiliations:** 1 State Key Laboratory for Innovation and Transformation of Luobing Theory, Key Laboratory of Cardiovascular Remodeling and Function Research of MOE, NHC, CAMS and Shandong Province, Department of Cardiology, Qilu Hospital of Shandong University, Jinan, China.; 2 Department of Critical Care Medicine, Qilu Hospital, Innovation Research Center for Sepsis and Multiple Organ Injury, Shandong University, Jinan, China.; 3 Department of Organ Transplantation, Qilu Hospital, and; 4 Department of Geriatric Medicine, Qilu Hospital, Shandong University, Jinan, China.; 5 Department of Critical Care Medicine, Shandong Provincial Hospital, Jinan, Shandong, China.; 6 Department of Cardiology, Shandong Provincial Qianfoshan Hospital, Jinan, Shandong, China.; 7 Department of Emergency, Qilu Hospital, Shandong University, Jinan, China.; 8 Department of Critical Care Medicine, Affiliated Tengzhou Hospital of Xuzhou Medical University/Tengzhou Central People’s Hospital, Shandong, China.

**Keywords:** Cardiology, Vascular biology, Cardiovascular disease, Ubiquitin-proteosome system

## Abstract

The osteogenic environment promotes vascular calcium phosphate deposition and aggregation of unfolded and misfolded proteins, resulting in ER stress in chronic kidney disease (CKD). Controlling ER stress through genetic intervention is a promising approach for treating vascular calcification. In this study, we demonstrated a positive correlation between ER stress–induced tribble homolog 3 (*TRIB3*) expression and progression of vascular calcification in human and rodent CKD. Increased *TRIB3* expression promoted vascular smooth muscle cell (VSMC) calcification by interacting with the C2 domain of the E3 ubiquitin-protein ligase Smurf1, facilitating its K48-related self-ubiquitination at Lys381 and Lys383 and subsequent dissociation from the plasma membrane and nuclei. This degeneration of Smurf1 accelerated the stabilization of the osteogenic transcription factors RUNX family transcription factor 2 (Runx2) and SMAD family member 1 (Smad1). C/EBP homologous protein and activating transcription factor 4 are upstream transcription factors of *TRIB3* in an osteogenic environment. Genetic KO of *TRIB3* or rescue of Smurf1 ameliorated VSMC and vascular calcification by stabilizing Smurf1 and enhancing the degradation of Runx2 and Smad1. Our findings shed light on the vital role of *TRIB3* as a scaffold in ER stress and vascular calcification and offer a potential therapeutic option for CKD.

## Abstract 2

TRIB3 mediates vascular calcification through facilitating self-ubiquitination and dissociation of Smurf1 in chronic renal disease

## Graphical abstract

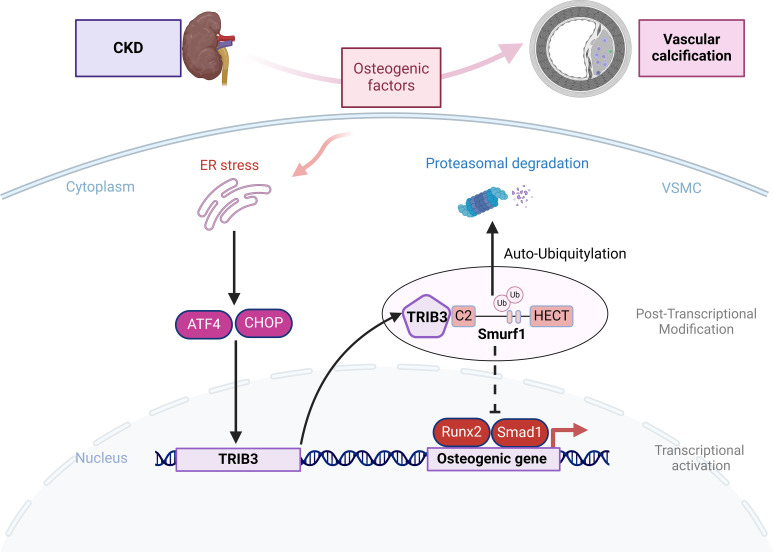

## Introduction

Vascular calcification, characterized by precipitation of hydroxyapatite mineral within the arterial wall, is an independent risk factor associated with cardiovascular diseases and cardiovascular mortality in both the general population and individuals with chronic kidney disease (CKD) (1). The prevalence of vascular calcification in CKD ranges from 47% to 92% (2, 3). In a significant number of patients with CKD, the size of coronary artery calcification is an independent risk factor and inversely correlated with estimated glomerular filtration rate (4). Traditional cardiovascular and CKD risk factors (diabetes, dyslipidemia, inflammation) (5) and related injury (phosphate [Pi] retention, excessive calcium intake, and dialysis experience) (6) are associated with the severity and progression of vascular calcification.

Calcification occurs in the arterial intima and media, with the vascular smooth muscle cell (VSMC) being a key cell type involved in the calcification process (7). In normal adult tissues, VSMCs exhibit a contractile phenotype characterized by a slow rate of proliferation and a predominant contractile function (8). In contrast to other muscle cells, VSMCs do not terminally differentiate and display phenotypic plasticity (9). These cells can respond to local and systemic stress, altering their phenotype. In an osteogenic environment, such as one rich in high Pi and bone morphogenetic protein (BMP), VSMCs are capable of altering their gene expression profile, enhancing the expression of osteoblastic markers such as RUNX family transcription factor 2 (*Runx2*) and SMAD family member 1 (*Smad1*), and resembling osteoblasts or chondrocytes (10).

The endoplasmic reticulum (ER) is the first organelle involved in protein synthesis, where most secretory and transmembrane proteins fold and mature (11). ER stress occurs when the accumulation of unfolded proteins exceeds the folding capacity of the ER, triggering the activation of the unfolded protein response (UPR) signaling pathway (12, 13). UPR and ER stress are prominently involved in various renal diseases, including primary glomerulonephritides (14), genetically associated glomerular diseases (15), diabetic nephropathy (16), acute kidney injury (AKI) (17), and CKD (18). In addition, it has been widely documented that ER stress can accompany vascular calcification in mice and rats (19). However, the mechanism linking vascular calcification and ER stress in CKD remains unclear.

Tribbles homolog 3 (*TRIB3*), a 45 kDa pseudokinase, was initially identified as a delayer of mitosis in *Drosophila* (20). Recently, *TRIB3* has been proven to be an ER stress and metabolic sensor involved in glucose and lipid metabolism and contributes to diabetes and related cardiovascular diseases (21). Our group found that insulin resistance and other ER stress markedly unregulated *TRIB3*, which serves as a scaffold for Akt and MAPKs (22). Data in our (22, 23) and other previous studies (24) proved that *TRIB3* expression is increased by CKD. Silencing *TRIB3* alleviates diabetic atherosclerosis (24) and nephropathy (24). However, the role of *TRIB3* in CKD-related vascular calcification remains unclear.

## Results

### Elevation of TRIB3 expression and ER stress during calcifying conditions.

**Figure 1:**
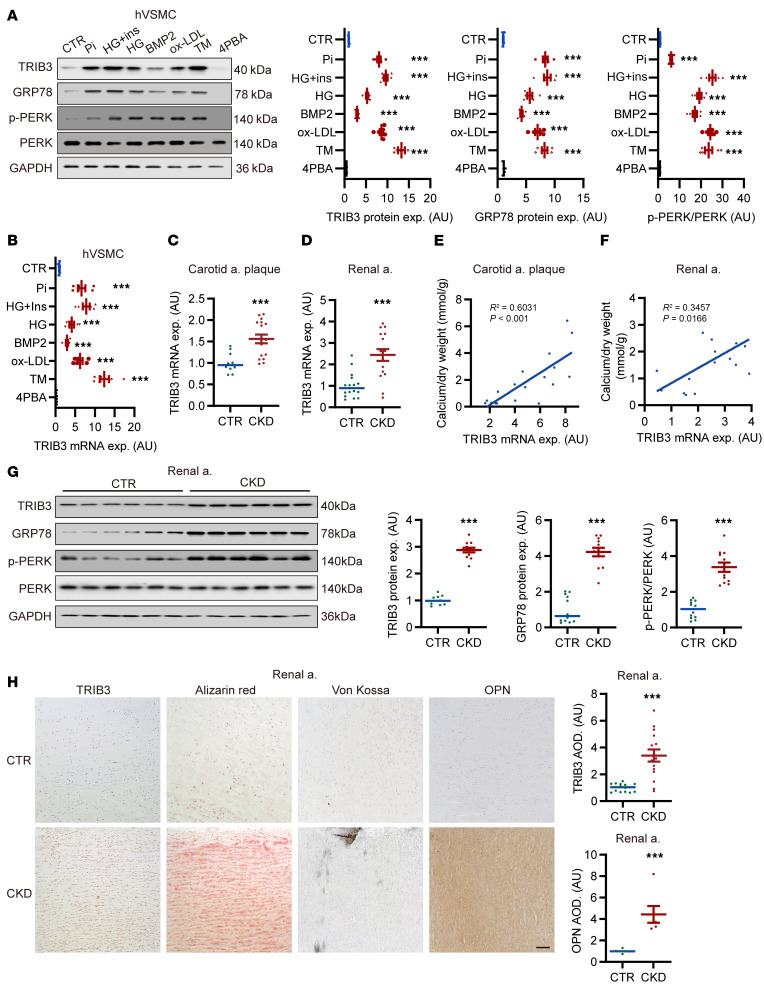
Elevated VSMC TRIB3 expression in ER stress. (**A**) Representative Western blots and analysis of TRIB3, GPR78, and phosphorylation of PERK expression in primary hVSMCs following treatment with L-glucose osmotic medium control (CTR) or 2.6 mM phosphate (Pi), 25 mM glucose plus 10 nM insulin (HG+Ins), 25 mM glucose (HG), 10 nM human bone morphogenetic protein 2 (BMP2), 80 μg/mL ox-LDL, 0.1 μg/mL tunicamycin, 5 μM 4-phenylbutyric acid (4PBA). *n* = 6 per group. Quantification of the protein expression of TRIB3, GPR78, and phosphorylation of PERK. (**B**) RT-qPCR analysis of TRIB3 relative mRNA expression in hVSMCs following indicated treatment. *n* = 6 per group. (**C** and **D**) RT-qPCR analysis of TRIB3 relative mRNA expression in carotid and renal arteries of patients with chronic kidney disease (CKD). *n* = 10–17 per group. (**E** and **F**) Correlation of TRIB3 relative mRNA expression and calcium deposition in CTR carotid and renal artery plaque tissue and that from patients with CKD. *P* represents the 2-tailed probability value of the Pearson’s correlation. *n* = 18 per group. (**G**) Representative Western blots and analysis of TRIB3, GPR78, and PERK protein expression in renal arteries from patients with CKD and individuals with normal renal function (*n* = 12 in each). (**H**) Representative original histological images and IHC analysis showing TRIB3 expression and ectopic calcification in CTR renal arteries and those from patients with CKD (*n* = 15 per group). alizarin red staining identifies mid-to-late-stage mineralization, Von Kossa staining identifies late-stage calcification, and osteopontin (OPN) (*n* = 6 per group) serves as a marker for osteogenic differentiation. Scale bar: 100 μm. Data are shown in scatter dot plots and as the arithmetic mean ± SEM (AU). Throughout the figure, relative values were compared against those of the CTR group. Statistical analyses were performed using 1-way ANOVA (**A** and **B**) and unpaired 2-tailed Student’s *t* test with Welch correction (**C**, **D**, **G**, and **H**). ***P < 0.001, statistically significant vs. CTR.

To investigate the effects of known osteogenic differentiation and ER stress triggers in VSMCs on vascular TRIB3 expression, we conducted a series of in vitro experiments. As shown in Figure 1, A and B, treatment with Pi, high glucose plus insulin, high glucose, recombinant BMP2 protein, oxidized LDL (ox-LDL), and the ER stress agonist tunicamycin substantially upregulated TRIB3 protein expression and mRNA transcription in human VSMCs (hVSMCs). These effects were paralleled by increased protein expression of ER stress markers (glucose-regulated protein 78 [GRP78] and phosphorylation of protein kinase R-like ER kinase [PERK]) but were abolished by the ER stress antagonist 4-phenylbutyric acid (4PBA). Further experiments were conducted to investigate TRIB3 expression in humans under uremic conditions. As shown in Supplemental Figure 1A (supplemental material available online with this article; https://doi.org/10.1172/JCI175972DS1), TRIB3 and ER stress markers were markedly upregulated following treatment with uremic sera (Supplemental Table 1).

In human arterial specimens (Supplemental Tables 2–4), atherosclerosis of the coronary and carotid arteries is represented by intimal layer calcification, whereas medial calcification occurs in the aorta. The mRNA expression of *TRIB3* was higher in the carotid, renal, and coronary arteries of patients with CKD compared with individuals with normal kidney function (Figure 1, C and D, and Supplemental Figure 1B). More critically, TRIB3 mRNA transcription and protein expression in different arteries of patients with CKD was substantially correlated with calcium deposition (Figure 1, E and F, and Supplemental Figure 1, C–E). Furthermore, TRIB3 protein levels were substantially elevated in renal artery samples from patients with CKD compared with individuals with normal renal function, and TRIB3 expression positively correlated with ER stress markers (Figure 1G and Supplemental Figure 1, F and G).

As demonstrated by histological examination, the TRIB3 protein levels and the osteogenic marker osteopontin (OPN) were elevated in the calcified intima and media of human arteries in patients with CKD compared with individuals acting as controls and were confined to the calcified arterial regions (Figure 1H and Supplemental Figure 1, H and I).

Furthermore, in mouse CKD models derived from acute kidney injury (unilateral ureteral obstruction mice, 5/6 nephrectomy mice) and chronic metabolic disturbance-related CKD (*db/db* mice, apolipoprotein E [*ApoE*] KO with streptozotocin [STZ] administration mice), *TRIB3* mRNA expression in the aorta was substantially higher than that in Sham or WT mice (Supplemental Figure 1, J–M).

### TRIB3 suppresses Runx2 and Smad1 degradation in VSMCs.

**Figure 2:**
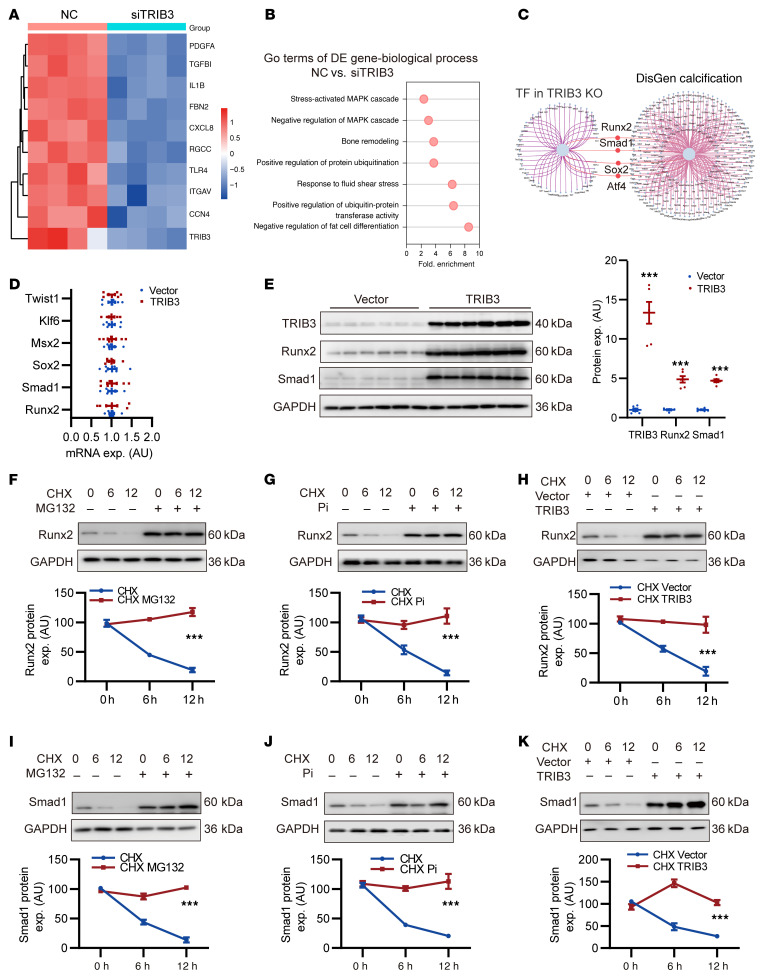
TRIB3 suppresses Runx2 and Smad1 degradation in VSMCs. (**A**) Heatmap of differentially expressed genes (DEGs) of vascular calcification phenotype gene cluster (DISGENET C0342649) in hVSMCs transfected with negative control (NC) or siTRIB3. (**B**) Gene ontology (GO) analysis of DEGs in hVSMCs transfected with NC or siTRIB3. (**C**) The overlap of transcription factor (TF) binding signature analysis for DEGs after TRIB3 KO and DISGENET C0342649. (**D**) RT-qPCR analysis of osteogenic TF (Runx2, Smad1, Sox2, Msx2, Klf6, and Twist1) mRNA expression in hVSMCs transfected with blank vector (Vector) and TRIB3 overexpression adenovirus (TRIB3). *n* = 6 per group. Statistical analyses were performed using the unpaired 2-tailed Student’s *t* test. Relative values were compared against those of the vector group. (**E**) Representative Western blots and analysis of protein expression in TRIB3, Runx2, and Smad1 in hVSMCs transfected with vector and TRIB3. *n* = 6 per group. Statistical analyses were performed using the unpaired 2-tailed Student’s *t* test. Relative values were compared against those of the vector group. ***P < 0.001, statistically significant vs. vector. (**F**–**K**) Representative Western blots and analysis of Runx2 (**F**–**H**) and Smad1 (**I**–**K**) protein expression in hVSMCs following treatment with or without MG132 (10 μM, 4 h), Pi (2.6 mM, 48 h), vector, or TRIB3 (48 h) and incubation with CHX (50 μg/mL) for indicated time points. *n* = 6 per group. CHX, cycloheximide. Statistical analyses were performed using repeated measures 2-way ANOVA. Data are shown in scatter dot plots and as the arithmetic mean ± SEM (AU). ***P < 0.001, statistically significant vs. CHX or CHX vector.

To investigate TRIB3 downstream biological functions and signaling pathways involved in VSMC calcification, we assessed the transcriptomes of hVSMCs treated with *TRIB3* siRNA and primary mouse VSMCs (mVSMCs) derived from *TRIB3*-KO mice using bulk RNA-Seq. Gene ontology (GO) analysis revealed that *TRIB3* was involved in bone remodeling and ubiquitin-dependent protein catabolic processes, except for classical metabolism regulation and the MAPK pathway in hVSMCs and mVSMCs (Figure 2, A and B, and Supplemental Figure 2, A and B). These data suggested that *TRIB3* participates in the regulation of osteogenic transcription factors and ubiquitination.

To identify upstream molecules associated with DEGs after *TRIB3* KO, transcription factor–binding signature analysis in KnockTF2.0 (25) was performed and overlapped with DISGENET C0342649 (https://disgenet.com/search?view=DISEASES&idents=C0342649&source=ALL&tab=GDA). We found that the functions of the osteogenic transcription factors *Runx2* and *Smad1* were downregulated after *TRIB3* KO (Figure 2C).

To validate the function of these osteogenic transcription factors in the elevation of *TRIB3* expression by simulating an osteogenic environment, *TRIB3* adenovirus was transfected into hVSMCs. Unexpectedly, *TRIB3* overexpression did not affect the transcription factors *Runx2*, *Smad1*, *Sox2*, Msh homeobox 2 (*Msx2*), the classical osteogenic Kruppel-like factor 6 (*Klf6*), or twist family BHLH transcription factor 1 (*Twist1*) transcript levels (Figure 2D). However, *TRIB3* overexpression increased Runx2 and Smad1 protein levels (Figure 2E). This suggests that TRIB3 regulates Runx2 and Smad1 protein levels posttranslationally.

Subsequently, we evaluated the stability of Runx2 and Smad1 using the proteasome inhibitor MG132 and the protein synthesis inhibitor cycloheximide (CHX). Runx2 and Smad1 protein levels substantially decreased in hVSMCs treated with CHX, and MG132 attenuated this effect. Pi treatment and *TRIB3* overexpression had an effect similar to that of MG132 in blocking Runx2 and Smad1 turnover (Figure 2, F–K). Thus, Runx2 and Smad1 are rapidly degraded through the proteasomal pathway in VSMCs. TRIB3 might increase Runx2 and Smad1 protein accumulation by suppressing proteasomal degradation in response to osteogenic stimuli.

### TRIB3 promotes Runx2 and Smad1 ubiquitination via interacting with Smurf1.

**Figure 3:**
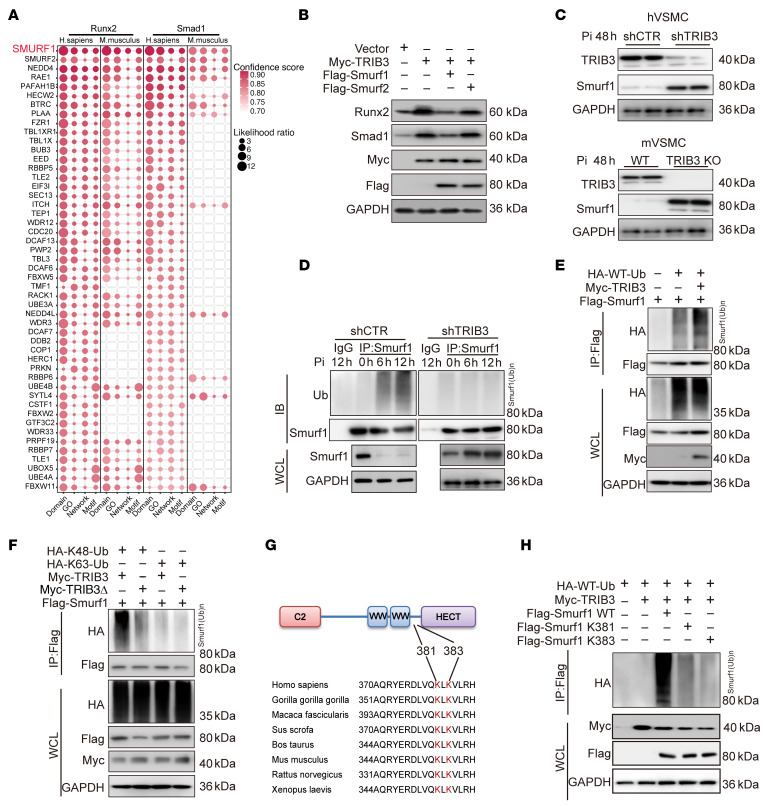
TRIB3 promotes Runx2 and Smad1 ubiquitination via interacting with Smurf1. (**A**) Matrix bubble diagram of E3 ubiquitin ligases prediction of Runx2 and Smad1 in human and mouse. (**B**) Representative Western blots of protein expression in Runx2 and Smad1 in hVSMCs transfected with exogenous TRIB3, Smurf1, and Smurf2 plasmid. The presented results represent 1 of 3 independent replicates. (**C**) Representative Western blots of protein expression in TRIB3 and Smurf1 in hVSMCs and mVSMCs treated with Pi (2.6 mM, 48 h). The presented results represent 1 of 3 independent replicates. (**D**) Representative Western blots of ubiquitination of Smurf1 in hVSMCs transfected with shTRIB3 and treated with Pi (2.6 mM) at the indicated times. The presented results represent 1 of 3 independent replicates. (**E**) Representative Western blots of ubiquitination of exogenous Smurf1 in HEK293T cells. The presented results represent 1 of 3 independent replicates. (**F**) Representative Western blots of ubiquitination of exogenous Smurf1 in HEK293T cells transfected with plasmids encoding the mutant of TRIB3 238–266aa (TRIB3Δ), HA-ubiquitin (K48), and HA-ubiquitin (K63). The presented results represent 1 of 3 independent replicates. (**G**) The multiple sequence alignment and K48 ubiquitin site prediction for Smurf1. (**H**) Representative Western blots of Smurf1 ubiquitination assay with exogenous Smurf1 (Smurf1 WT), Smurf1 mutant with the lysine residue replaced by arginine in K381 (K381R), and K383 (K383R). The presented results represent 1 of 3 independent replicates.

Although *TRIB3* suppresses proteasomal degradation of Runx2 and Smad1, TRIB3 does not belong to any of the ubiquitination or deubiquitination enzyme families. E3 ubiquitin ligases are crucial for catalyzing the ubiquitination and facilitating the transfer of ubiquitin (26). Therefore, we examined whether TRIB3 regulates Runx2 and Smad1 via intermediary E3 ubiquitin ligases. We found that Runx2 and Smad1 share a common E3 ubiquitin ligase, which is the Smad ubiquitination regulatory factor 1 (Smurf1), in humans and mice via Ubibrowser 2.0, usingcomprehensive protein motif analysis, protein GO, and network annotation (Figure 3A). Because Smurf2 derived from the same family also showed high confidence score and likelihood ratio in Ubibrowser 2.0, we separately transfected exogenous Smurf1 and Smurf2 into hVSMCs overexpressing *TRIB3*. The results revealed that only Smurf1 could reverse the increase in Runx2 and Smad1 proteins induced by TRIB3 (Figure 3B). Indeed, TRIB3 deficiency in human or mVSMCs resulted in endogenous smurf1 accumulation in response to Pi stimuli (Figure 3C).

Smurf1 belongs to the neural precursor cell–expressed developmentally downregulated protein 4 (NEDD4) subfamily and regulates ligase activity-independent self-ubiquitylation (27). In addition, TRIB3 time-dependently increased endogenous smurf1 self-ubiquitination and proteasomal turnover in hVSMCs (Figure 3D). *TRIB3* knockdown could decrease endogenous smurf1 self-ubiquitination (Figure 3D). To confirm this finding, we transfected exogenous *TRIB3* and *Smurf1* into HEK293T cells, which confirmed the regulatory effect of TRIB3 on Smurf1 ubiquitination (Figure 3E). A mutant of TRIB3 238–266aa was used to confirm the interaction site with Smurf1 (Figure 3F), as reported previously (28). It was confirmed that TRIB3 promoted Smurf1 self-ubiquitination via K48-linked, but not K63-linked, ubiquitination (Figure 3F). Multiple sequence alignments and ubiquitination site prediction indicated that TRIB3-mediated K48-linked ubiquitination may occur at the highly conserved K381 and K383 sites of Smurf1 (Figure 3G). We then created a Smurf1 mutant where a lysine residue was substituted with arginine and assessed the polyubiquitination level of this mutant in HEK293T cells. In cells expressing Smurf1, whose K381R or K383R residue was replaced, the effect of TRIB3 on accelerating Smurf1 ubiquitination was attenuated (Figure 3H).

### TRIB3 interacted Smurf1 and promoted its self-ubiquitination through the C2 domain.

**Figure 4:**
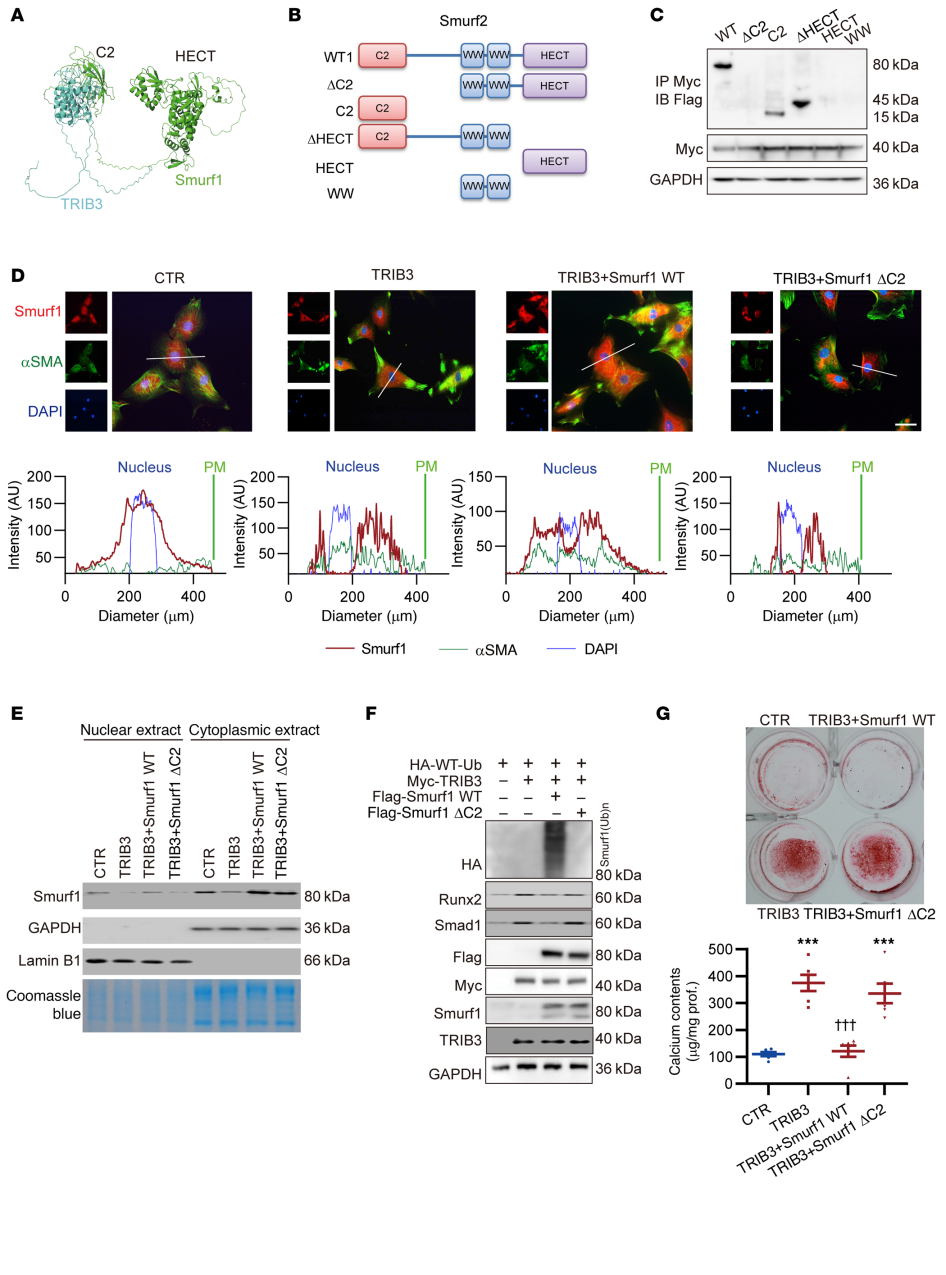
TRIB3 interacted Smurf1 and promoted its self-ubiquitination through C2 domain. (**A**) Structure docking to simulate the interaction between Smurf1 and TRIB3. (**B**) Smurf1 domain structure and deletion mutants used in the study (WT, ΔC2, C2, ΔHECT, HECT, and WW). (**C**) Representative IP Western blots of Myc-TRIB3 and Flag-Smurf1 and mutants in HEK293T cells were transfected with plasmid for 48 hours. The presented results represent 1 of 3 independent replicates. (**D**) Representative immunofluorescence of Smurf1 and αSMA in hVSMCs transfected with TRIB3, Smurf1 WT, and Smurf1ΔC2 for 48 hours. The DAPI indicated the nuclei, and the margin of αSMA indicated the plasma membrane (PM). The white line is the path of the colocalization analysis. Scale bar: 20 μm. The presented results represent 1 of 3 independent replicates. (**E**) Representative Western blots of cellular fractionation showing Smurf1 expression in the nuclear and cytoplasmic fractions in hVSMCs. The presented results represent 1 of 3 independent replicates. (**F**) Representative Western blots of ubiquitination of exogenous Smurf1 and Smurf1ΔC2 with TRIB3 in HEK293T cells. The presented results represent 1 of 3 independent replicates. (**G**) Representative alizarin red staining of hVSMCs transfected with plasmids encoding mutant of Smurf1, Smurf1ΔC2, and TRIB3 and then treated with Pi (2.6 mM) for 7 days (*n* = 6 in each). The calcium contents analysis of hVSMCs transfected with plasmids encoding mutant of Smurf1, Smurf1ΔC2, and TRIB3 and then treated with Pi (2.6 mM) for 7 days. Statistical analyses were performed using 2-way ANOVA. Relative values were compared against those of the CTR group. ***P < 0.001, statistically significant vs. CTR. ^†††^*P* < 0.001, statistically significant vs. TRIB3. Each experiment was repeated independently for a minimum 3 times. Data are shown in scatter dot plots and as the arithmetic mean ± SEM (AU).

TRIB3 exerts various effects through interactions with other proteins (29). A previous study (28) identified amino acids 238–266 of TRIB3 as key regions interacting with Smurf1. Nonetheless, the domain in which Smurf1 interacted with TRIB3 remains unclear. Using molecular docking and deletion mutants of the Smurf1 domain, we observed that TRIB3 interacts with the C2 domain of Smurf1 (Figure 4, A–C). In the molecular dynamics simulations performed using Gromacs, the TRIB3 and Smurf1 complex remained stable, as indicated by the stable root-mean-square deviation after 20,000 ps simulation, suggesting a slow but stable interaction between TRIB3 and Smurf1 (Supplemental Figure 3A). Additionally, upon binding, the homologous to the E6-AP carboxyl terminus (HECT) domain of Smurf1 exhibited conformational unfolding (Supplemental Video 1), indicated by two low energy regions in the energy landscape (Supplemental Figure 3B). Through surface plasmon resonance (SPR), the equilibrium dissociation constant (KD) between the two recombinant proteins was determined to be 1.03 × 10^–6^ M (Supplemental Figure 3C). Given that the C2 domain is crucial for Smurf1 localization to the plasma membrane (28), we investigated whether the subcellular localization of Smurf1 is affected by its interaction with TRIB3. Endogenous Smurf1 was localized in whole cells from the plasma membrane to the nucleus, but exogenous TRIB3 resulted in Smurf1 dissociation from the plasma membrane and nucleus and conglomeration in the cytoplasm. Transfection with exogenous full-length *Smurf1* (Smurf1 WT) alleviated TRIB3-induced dissociation from the plasma membrane and nucleus, and some Smurf1 was localized to the plasma membrane and nucleus. However, the mutant Smurf1 lacking C2 domain (Smurf1 ΔC2) showed none of these effects (Figure 4D). Additional cellular fractionation by Western blotting yielded similar results (Figure 4E). Furthermore, exogenous TRIB3 did not induce the promotion of ubiquitination for Smurf1 ΔC2 in HEK293T cells, and exogenous Smurf1ΔC2 did not reverse TRIB3-induced elevation of Runx2 and Smad1 (Figure 4F). Additionally, we verified that the introduction of exogenous Smurf1 WT successfully reversed the accelerated calcification of hVSMCs induced by *TRIB3*. However, Smurf1 ΔC2 had no such effect (Figure 4G).

Our data collectively demonstrated that TRIB3 interacts with the Smurf1 C2 domain to promote its dissociation from the plasma membrane and nucleus, translocates into the cytoplasm for self-ubiquitination, and promotes calcification in VSMCs.

### ATF4 and CHOP induced TRIB3 transcription in Pi stimuli.

**Figure 5:**
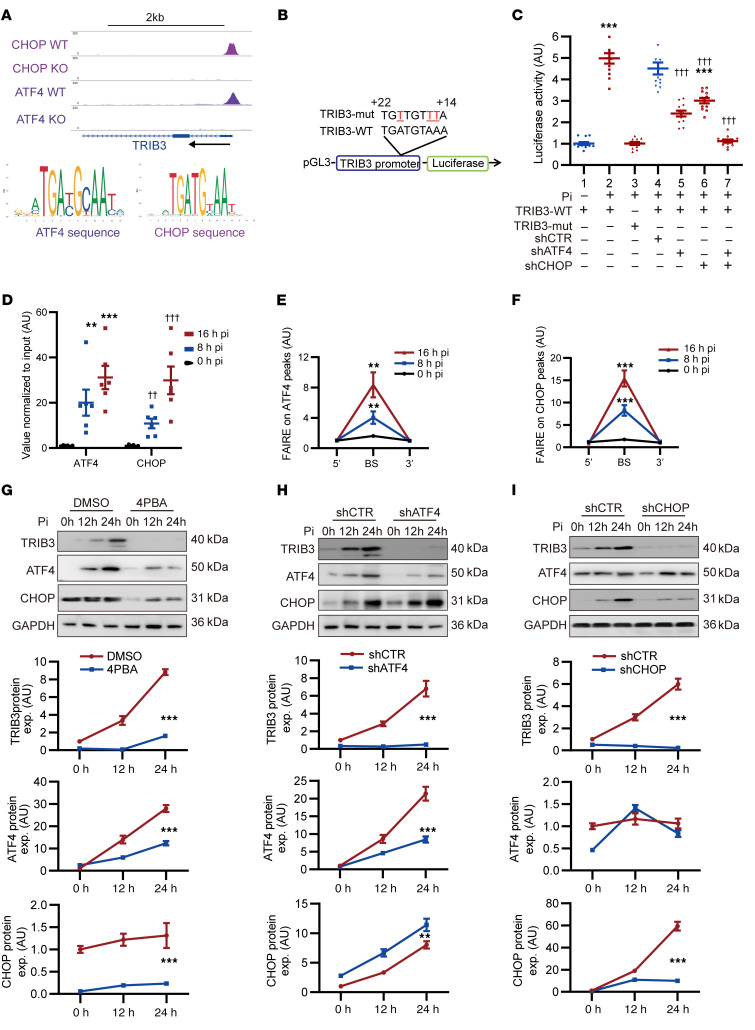
ATF4 and CHOP induced TRIB3 transcription in phosphorate stimuli. (**A**) IGV was used to display data for the TRIB3 loci, with ChIP-Seq tracks and sequence logo for ATF4 and CHOP. (**B**) Wild-type or mutant TRIB3 promoter luciferase plasmid. (**C**) (41.Luciferase activity of mVSMCs transfected with the TRIB3-WT or TRIB3-mut promoter luciferase plasmid and treated with Pi (2.6 mM) for 12 h or transfected with shRNA to knock down ATF4 or CHOP for 48 h). Statistical analyses were performed using 2-way ANOVA. ***P < 0.001, statistically significant vs. 1.TRIB3-WT without Pi treatment; ^†††^*P* < 0.001, statistically significant vs. 4.transfected with shCTR and TRIB3-WT with Pi treatment. (**D**) ChIP-PCR showing enrichment of both ATF4 and CHOP at the TRIB3 binding site (BS) in mVSMCs treated Pi (2.6 mM) for 24 hours. Statistical analyses were performed using 1-way ANOVA. **P < 0.01, ***P < 0.001, statistically significant vs. 0 h Pi treatment (ATF4); ^††^*P* < 0.01, ^†††^*P* < 0.001, statistically significant vs. 0 h Pi treatment (CHOP). (**E** and **F**) Formaldehyde-assisted isolation of regulatory elements–ChIP (FAIRE-ChIP) PCR was performed on Pi (2.6 mM) for indicated times in mVSMCs. Statistical analyses were performed using repeated measures 2-way ANOVA. (**G**–**I**) Representative Western blots of TRIB3, ATF4, and CHOP in mVSMCs treated with Pi (2.6 mM) at the indicated times after pretreatment with 4PBA 5 μM for 12 hours or transfection with shRNA for knockdown of ATF4 and CHOP for 48 hours. Statistical analyses were performed using repeated measures 2-way ANOVA. Data are shown in scatter dot plots and as the arithmetic mean ± SEM (AU). *P < 0.05, **P < 0.01, ***P < 0.001, statistically significant vs. 0 h treatment, DMSO, or shCTR. Each experiment was repeated independently 3–6 times. Data are shown in scatter dot plots and as the arithmetic mean ± SEM (AU).

To investigate the mechanism underlying the increased transcription and expression of TRIB3 in response to Pi-induced calcification, we initially reanalyzed the data deposited at GEO (GSE35681), and confirmed the notable enrichment of ATF4 and C/EBP homologous protein (CHOP) in the same *TRIB3* promoter region under ER stress conditions in mouse embryonic fibroblasts. Moreover, ATF4 and CHOP shared highly similar sequences (Figure 5A). Therefore, we constructed a *TRIB3* promoter reporter luciferase plasmid and related core binding site mutant plasmids (*TRIB3*-WT and *TRIB3*-mut) (Figure 5B). Pi substantially boosted luciferase activity from the *TRIB3*-WT promoter-reporting plasmid but had no effect on *TRIB3*-mut. Additionally, ATF4 and CHOP knockdown effectively reduced or eliminated the Pi-induced increase in luciferase activity, respectively (Figure 5C).

After ER stress stimulation, mVSMCs exhibited increased binding of the *TRIB3* promoter to ATF4 and CHOP, as determined by ChIP-PCR in Pi-treated cells (Figure 5D). Formaldehyde-assisted isolation of regulatory elements–PCR (FAIRE-PCR) confirmed the binding of ATF4 and CHOP to the open chromatin region of the *TRIB3* promoter (Figure 5, E and F).

Furthermore, the ER stress inhibitor 4PBA suppressed the upregulation of TRIB3 caused by Pi while simultaneously inhibiting the transcription of *ATF4* and *CHOP* (Figure 5G). Additionally, the knockdown of *ATF4* and *CHOP* attenuated the Pi-induced increase in TRIB3 expression (Figure 5, H and I). To confirm that the upregulation of TRIB3 was driven by ER stress, we treated mVSMCs with the ER stress agonist tunicamycin, which yielded similar results (Supplemental Figure 4).

Taken together, these findings indicate that Pi promotes the transcriptional upregulation of *TRIB3* via the facilitation of ATF4 and CHOP binding, potentially serving as a mechanism for the promotion of TRIB3 expression in a calcified environment.

### Effect of TRIB3 deficiency on ER-induced osteogenic differentiation of primary aortic smooth muscle cells.

**Figure 6:**
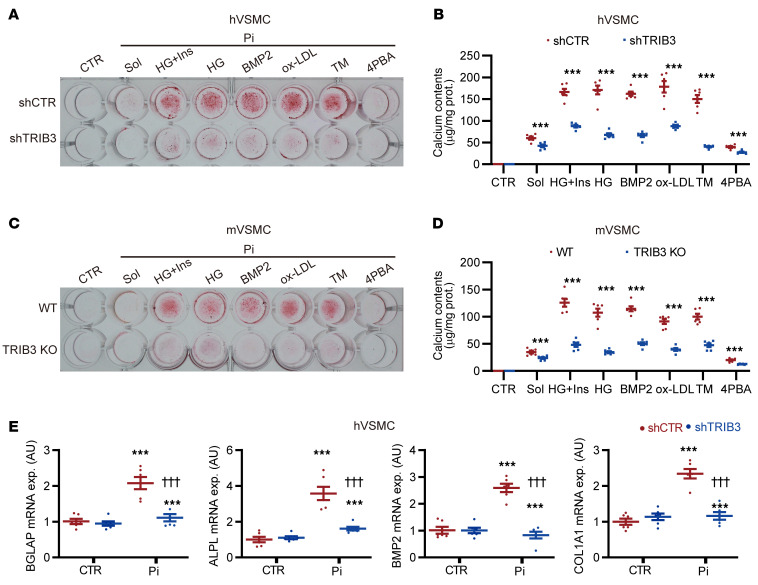
Effect of TRIB3 deficiency on ER-induced osteogenic transdifferentiation of primary aortic smooth muscle cells. (**A**) Representative alizarin red staining of hVSMCs transfected with shCTR and shTRIB3 plasmids and then treated with L-glucose osmotic medium control (CTR), solvent (Sol), 2.6 mM Pi plus 25 mM HG plus 10 nM Ins, 25 mM HG, 10 nM BMP2, 80 μg/mL ox-LDL, 0.1 μg/mL TM, or 5 μM 4PBA for 7 days. (**B**) Calcium content analysis for **A** (*n* = 6 in each). Statistical analyses were performed using 1-way ANOVA. Relative values were compared against those of the CTR group. (**C**) Representative alizarin red staining of mVSMCs from WT and TRIB3-KO mice that were then treated with 2.6 mM Pi plus 25 mM HG plus 10 nM Ins, 25 mM HG, 10 nM BMP2, 80 μg/mL ox-LDL, 0.1 μg/mL TM, or 5 μM 4PBA for 7 days. (**D**) Calcium content analysis for **C** (*n* = 6 in each). Statistical analyses were performed using 1-way ANOVA. Relative values were compared against those of the CTR group. (**E**) RT-qPCR analysis of osteogenic factor (BGLAP, ALPL, BMP2, and COL1A1) mRNA expression in hVSMCs transfected with shCTR and shTRIB3 plasmids and then treated with Pi (2.6 mM, 7d) (*n* = 6 in each). Statistical analyses were performed using 2-way ANOVA. ***P < 0.001, statistically significant vs. CTR or Sol; ^†††^*P* < 0.001, statistically significant vs. Pi plus shCTR. Data are shown in scatter dot plots and as the arithmetic mean ± SEM (AU).

To further investigate the function of *TRIB3* in the osteogenic differentiation and calcification of VSMCs under ER stress conditions, hVSMCs and mVSMCs were differentiated in Pi combined with high glucose plus insulin, high glucose, recombinant BMP2 protein, ox-LDL, tunicamycin, and 4PBA. Through shRNA silencing of *TRIB3* in hVSMCs or KO of *TRIB3* in mVSMCs, both exhibited resistance to ER stress–induced calcification compared with control shRNA-treated hVSMCs or WT mVSMCs (Figure 6, A–D). Furthermore, the downstream calcification factors of Runx2 and Smad1, including bone γ-carboxyglutamate protein (*BGLAP*), tissue nonspecific alkaline phosphatase (*ALPL*), *BMP2*, and collagen type I α 1 (*COL1A1*), were upregulated in response to Pi stimulation in hVSMCs. Importantly, this upregulation was reversed by the deficiency of *TRIB3* (Figure 6E).

### Effect of TRIB3 deficiency during AKI-induced CKD vascular calcification in mice.

**Figure 7:**
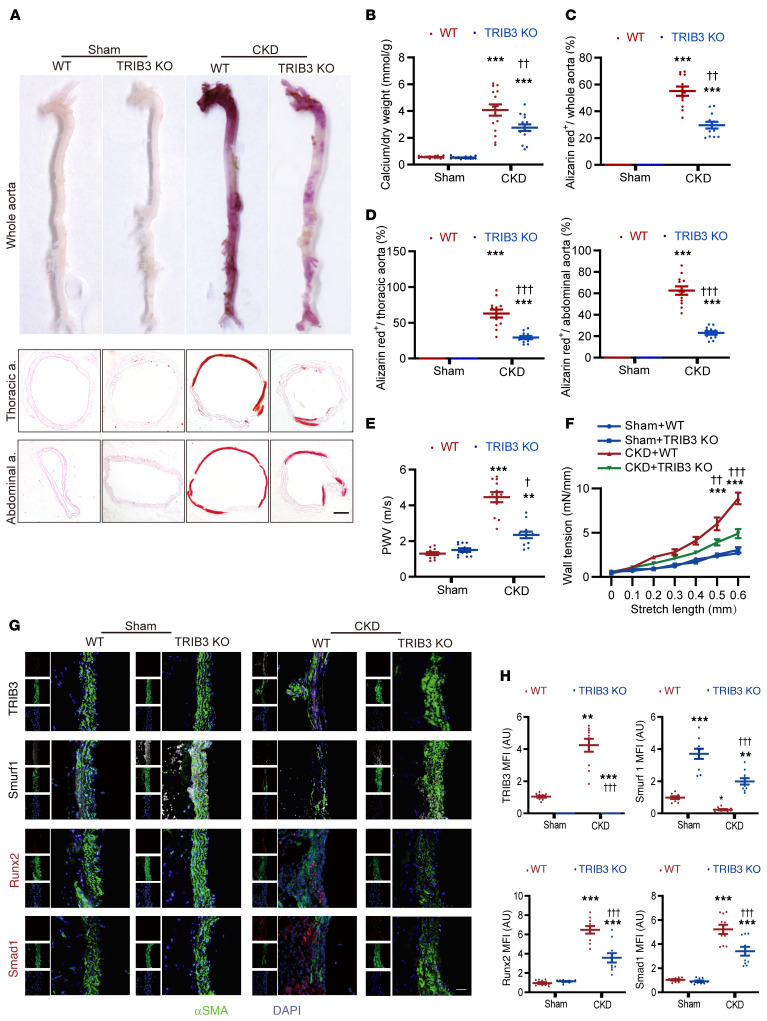
Effect of TRIB3 deficiency during acute kidney injury–induced CKD vascular calcification in mice. (**A**) Representative alizarin red staining of whole aorta and thoracic and abdominal aorta section images showing aortic alizarin red staining in acute kidney injury–induced (AKI-induced) CKD mice. Scale bar: 100 μm. Calcified areas are shown with red staining. (**B**) Calcium content analysis in the aortic arch of AKI-induced CKD mice, normalized by dry weight. Statistical analyses were performed using 2-way ANOVA. (**C** and **D**) The ratio of alizarin red–positive area to the whole aortic area, thoracic aorta, and abdominal aorta in the indicated group of mice (*n* = 12 in each). Statistical analyses were performed using 2-way ANOVA. (**E**) Abdominal aortic pulse wave velocity (PWV) in AKI-induced CKD mice (*n* = 12 in each). Statistical analyses were performed using 2-way ANOVA. (**F**) Wall tension (*n* = 3 rings, 6 mice per group; mN/mm) during mechanical stretch (mm) ex vivo of abdominal aorta isolated from AKI-induced CKD mice. (**G**) Representative immunofluorescence of TRIB3, Smurf1, Runx2, and Smad1 in thoracic aortic tissue of AKI-induced CKD mice. Scale bar: 50 μm. (**H**) The quantitative analysis for **G** (*n* = 5 incontinuous sections from 10 mice per group). Statistical analyses were performed using 2-way ANOVA. **P < 0.01, ***P < 0.001, statistically significant vs. sham WT mice; ^†^*P* < 0.05, ^††^*P* < 0.01, and ^†††^*P* < 0.001, statistically significant vs. CKD WT mice. *n* = 15 per group unless otherwise indicated. Data are shown in scatter dot plots and as the arithmetic mean ± SEM (AU).

To assess the in vivo relevance of *TRIB3* in medial vascular calcification, experiments were conducted in *TRIB3*-KO mice and corresponding WT mice in a mouse model of CKD after AKI. After 12 weeks of right cortical electrocautery and left total nephrectomy, plasma urea showed a 2- to 3-fold elevation in both *TRIB3*-KO and WT mice compared with the sham group (Supplemental Table 5). As shown by alizarin red staining and quantification of aortic calcium content, CKD mice showed severe aortic calcification. *TRIB3* KO reduced calcification in the aorta of CKD mice (Figure 7, A–D). In contrast, the aortic pulse wave velocity (PWV), an indicator of vascular rigidity, increased in CKD mice, but the PWV elevation effect was blunted in *TRIB3*-KO mice (Figure 7E). For ex vivo vascular mechanical index, wall tension was substantially increased in the aortic rings of CKD mice after 6 weeks. *TRIB3*-KO mice developed less wall tension after mechanical stretch than aortic rings from WT CKD mice (Figure 7F). The expression of the osteogenic transcription factors Runx2 and Smad1 increased in the aortic tissue of CKD mice, and these effects were substantially diminished in *TRIB3*-KO mice (Figure 7, G and H).

Additionally, we examined female CKD mice after AKI, and the results indicated that the above mechanisms are applicable to female individuals (Supplemental Figure 5). Using mouse models with vascular endothelial– or smooth muscle cell–specific *TRIB3* KO, we observed that *TRIB3* KO in smooth muscle cells resulted in a marked decrease in vascular calcification. In contrast, endothelial cell–specific *TRIB3*-KO mice did not exhibit this phenotype (Supplemental Figure 6).

Taken together, these data indicate that VSMC *TRIB3* promotes vascular tunica media calcification and stiffness by supporting Smad1 and Runx2 protein stability in a mouse model of CKD after AKI.

### Effect of TRIB3 deficiency on vascular calcification induced by metabolic CKD in mice.

**Figure 8:**
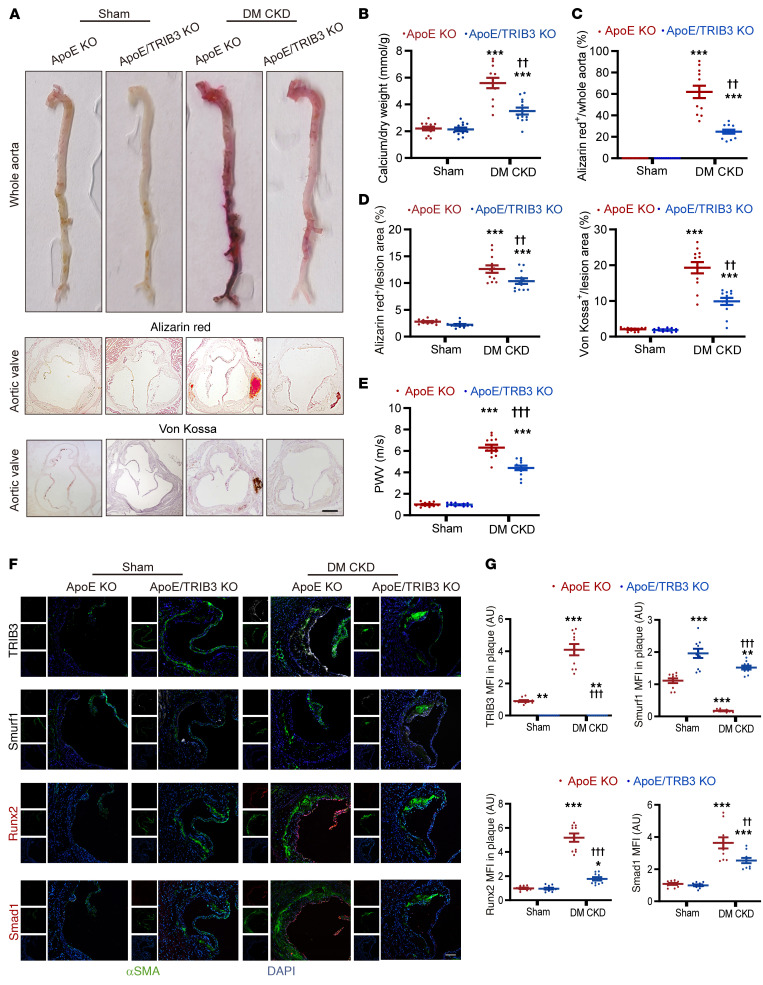
Effect of TRIB3 deficiency during metabolic CKD-induced vascular calcification in mice. (**A**) Representative alizarin red staining of whole aorta images, alizarin red staining of aortic valve sections, and Von Kossa staining of aortic valve sections in metabolic CKD mice. Scale bar: 100 μm. Calcified areas are shown with red staining. (**B**) Calcium content analysis in the aortic arch of metabolic CKD mice, normalized by dry weight. (**C**) The area ratio of calcification to the whole aortic area in metabolic CKD mice. Statistical analyses were performed using 2-way ANOVA. (**D**) The ratio of alizarin red–positive and Von Kossa–positive area to the whole aortic valves (sections with max valve plaque lesion area). Statistical analyses were performed using 2-way ANOVA. (**E**) PWV in metabolic CKD mice. Statistical analyses were performed using 2-way ANOVA. (**F**) Representative immunofluorescence of TRIB3, Smurf1, Runx2, and Smad1 in thoracic aortic tissue of metabolic CKD mice. Scale bar: 50 μm. (**G**) The quantitative analysis for **F** (*n* = 5 incontinuous sections from 10 mice per group). Statistical analyses were performed using 2-way ANOVA. **P < 0.01, ***P < 0.001, statistically significant vs. sham ApoE-KO mice; ^††^*P* < 0.01, ^†††^*P* < 0.001, statistically significant vs. diabetes mellitus. ApoE-KO mice. *n* = 12 per group unless otherwise indicated. Data are shown in scatter dot plots and as the arithmetic mean ± SEM (AU).

CKD-derived diabetes and other chronic metabolic diseases are widely recognized. Further experiments were conducted to examine the impact of *TRIB3* deficiency in a clinically relevant model of metabolic renal failure. A model of CKD combined with diabetes and hyperlipidemia was developed to investigate the role of *TRIB3* in metabolic CKD. Subtotal nephrectomy and STZ, combined with a diabetic diet, caused uremia in *ApoE*-KO mice and in double-KO *TRIB3* and *ApoE* mice (*ApoE*/*TRIB3* KO) (Supplemental Table 5). Histological staining confirmed podocyte injury and fibrosis in CKD model–related hyperlipidemia and hyperglycemia (Supplemental Figure 7). Calcium nodule formation substantially increased in the aortic roots of CKD mice, indicating intimal vascular calcification (Figure 8A). Similarly, in CKD after AKI, *TRIB3* KO substantially decreased calcium disposition in the aorta and aortic roots of patients with metabolic CKD (Figure 8, A–D). Furthermore, metabolic CKD substantially upregulated PWV (Figure 8E), and *TRIB3* KO substantially decreased these effects, although no significant difference was detected in arterial blood pressure among the groups of mice (Supplemental Table 5). In metabolic CKD mice, the expression of Smurf1 was suppressed, whereas the levels of Runx2 and Smad1 increase in the aortic roots. However, in *TRIB3*-KO mice, these effects were improved (Figure 8, F and G). To exclude the potential impact of *ApoE* KO, we investigated the effects of *TRIB3* KO alone on metabolic diabetic CKD model. Our findings demonstrate that *TRIB3* KO alleviates vascular calcification in diabetic metabolic CKD mice, showing similar results to the *ApoE-*KO metabolic CKD model (Supplemental Figure 8). Thus, *TRIB3* deficiency protects against vascular intimal calcification in metabolic CKD.

### Effect of TRIB3 deficiency on vascular calcification induced by high-dose vitamin D3 injection in mice.

Some patients with vascular calcification do not have CKD. To explore the role of *TRIB3* in the absence of CKD, we employed a high-dose vitamin D3 injection model to simulate acute vascular calcification induced by elevated calcium and Pi. The results also indicated that *TRIB3* substantially attenuated vitamin D3–induced vascular calcification (Supplemental Figure 9). These findings suggest the broader role of *TRIB3* in modulating vascular calcification.

## Discussion

*TRIB3* is a crucial scaffold protein that contributes to the regulation of cellular stress, differentiation, and glucose-lipid metabolism. Previous studies have implicated *TRIB3* signaling in diabetic nephropathy and atherosclerotic vascular diseases. However, its role in the progression of vascular calcification in CKD is insufficiently elucidated. This study sheds light on the involvement of VSMC *TRIB3* as a sensor of ER stress during CKD-associated vascular disease progression, leading to the enhanced stabilization of osteogenic transcription factors and subsequent vascular calcification.

Vascular calcification plays a significant role in cardiovascular mortality and poses a greater threat to patients with CKD and atherosclerotic plaques in arteries (7). Increasing evidence suggests that CKD-related risk factors and complications, such as insulin resistance, dyslipidemia, hypercalcemia, and hyperphosphatemia, impose cellular stress, triggering UPR and ER stress (19). However, the current state of research fails to elucidate the unification of diverse calcification factors in CKD, specifically, how they collectively converge through ER stress to induce vascular calcification. Our study introduces *TRIB3* as a pivotal scaffold protein at the intersection of ER stress and ubiquitination, thereby establishing a paradigm connecting CKD-associated ER stress with the ubiquitination of various calcification factors, ultimately leading to vascular calcification.

During ER stress, GRP78 dissociates from the ER, subsequently activating downstream transcription factors ATF4 and CHOP through phosphorylation or transcriptional mechanisms (19). Our reanalysis of CHOP and ATF4 ChIP-Seq data indicated that *TRIB3* is a shared target gene of CHOP and ATF4. Further data confirmed that osteogenic conditions induced ER stress in VSMCs, resulting in increased *TRIB3* transcription driven by CHOP and ATF4, highlighting the important role of *TRIB3* as an effector in ER stress triggered by CKD in osteogenic environments.

*TRIB3* functions as a pseudokinase during the integrated stress response, directly binding to the “Thr-308” phosphorylation on AKT1 and inhibiting its activation (30). On the other hand, *TRIB3* can interact with ubiquitin protein binding ligases to regulate the stability of substrate proteins (30, 31). In our study, transcriptomic data suggested that *TRIB3* regulates the ubiquitination process in VSMCs. Further experiments confirmed that *TRIB3* promotes the ubiquitination of Smurf1 in osteogenic environments. This finding is consistent with previous results in Cos7 cells (monkey kidney fibroblast-like cells) (28) but is contrary to the results observed in the MDA-MB-231 cell line (human breast cancer cell line) (27). These results indicate that the regulation of E3 ubiquitin ligase activity by *TRIB3* may exhibit cell selectivity.

A previous study found *TRIB3* interaction with the Smurf family: *TRIB3* interacts with Smurf2, triggering the degradation of SMAD3 (32) and *TRIB3* dissociates from bone morphogenetic protein receptor II and triggers degradation of Smurf1 (28). We confirmed the direct interaction between TRIB3 and Smurf1 as well as the promotion of self-ubiquitination in hVSMCs and mVSMCs. Several studies have documented the involvement of Smurf1-mediated K48- and K63-linked polyubiquitination (33). K48-linked ubiquitination promotes substrate protein degradation by the proteasome, whereas K63-linked polyubiquitin chains participate in proteasome-independent mechanisms (33). Using VSMCs, we demonstrated that TRIB3-mediated self-ubiquitination of Smurf1 in CKD occurs through a K48-dependent proteasomal pathway. Moreover, *TRIB3* accelerated Smurf1 self-ubiquitination with K48-linked ubiquitin chains at Lys381 and Lys383, resulting in enhanced translocalization and protein degeneration.

The self-ubiquitination level and intracellular localization of Smurf1 are modulated by the ER stress sensor *TRIB3*. Smurf1, composed of a C2 domain, 2 WW domains, and a HECT domain, is a critical regulator of TGF-β and BMP signaling (34). The C2 domain of Smurf1 interacts with the HECT domain of another Smurf1 and forms homodimers thereby inhibiting its self-ubiquitination (35, 36). In fact, our study revealed that TRIB3 interacts with the C2 domain of Smurf1, and molecular simulations speculated that TRIB3 binds Smurf1 C2 and lifts the HECT domain, triggering self-ubiquitination of Smurf1.

The Smurf1 C2 domain, known as the lipid-binding domain, is not only localized to the plasma membrane but also at negatively charged intracellular sites, serving as a selective binding domain (27). The C2 domain of Smurf1 exhibits substrate selection and cellular localization. Substrates of Smurf1 are localized to the plasma membrane (e.g., TGF-β receptors and RhoA), cytoplasm (Smad1/5, MEKK2), or nucleus (Smad1/5, JunB and Runx2) (37). Immunofluorescence studies demonstrated that TRIB3 binding to Smurf1 induced by Pi treatment or plasmid transfection resulted in Smurf1 detachment from the plasma membrane and nucleus, then causing cytoplasmic aggregation. This dissociation of Smurf1 caused by *TRIB3* may be one of the reasons for the altered ubiquitination levels of Smurf1 substrates such as Smad1 and Runx2. RhoA and Axin1, substrates of Smurf1 and part of the Wnt pathway, are linked to vascular calcification (38). However, *TRIB3* KO did not substantially reduce Wnt signaling during calcification (Supplemental Figure 10), suggesting that its role in this pathway requires further study.

ER stress is an active response of renal and vascular cells to survival stress (39). The emergence, during the evolution of pseudokinases, of TRIB3, which acts as scaffolding protein and inhibits the “real kinase” activity, assists cells in accomplishing complex functional transformations in the face of survival stress, including inhibiting proliferation, reducing protein synthesis, and increasing protein degradation (40). Either the need for renal repair after AKI or the need for metabolic transformation of the kidney in chronic metabolic disease drives cellular ER stress (18, 40). However, persistent calcification stimuli in CKD result in sustained expression of TRIB3 in VSMCs, turning on the additional function of *TRIB3* to accelerate Smurf1 ubiquitination, leading to irreversible differentiation of VSMCs toward osteoblasts and, ultimately, resulting in vascular calcification.

In conclusion, our data indicate that *TRIB3*, an ER stress sensor in VSMCs, tightly regulates the intracellular localization of the E3 ubiquitin ligase Smurf1 and its K48-related self-ubiquitination, subsequently controlling the transcription factor stability of Runx2 and Smad1. *TRIB3* acts as a scaffold molecule between ER stress and specific ubiquitin-mediated degradation of calcification transcription factors in CKD. Inhibition of TRIB3 may represent a potential strategy to counteract vascular calcification associated with CKD.

## Methods

### Sex as a biological variable.

Our study examined male and female animals, and similar findings are reported for both sexes.

### Animal protocols.

*TRIB3*-KO mice were a gift from Laurie J. Goodyear,Section on Integrative Physiology and Metabolism, Joslin Diabetes Center, Harvard Medical School, Boston, Massachusetts, USA, constructed as described previously (20). *Trib3*-floxp mice were purchased from GemPharmatech (strain no. T019128, C57BL/6JGpt-*Trib3em1Cflox*/Gpt). Homozygous *Trib3*-floxp mice were crossed with SM22-Cre and Cdh5-Cre mice to generate smooth muscle cell–specific *TRIB3*-KO (SMC-*TRIB3^KO^*) and endothelial cell–specific *TRIB3*-KO (EC-*TRIB3^KO^*) mice, respectively. Homozygous *Trib3*-floxp mice served as controls for *TRIB3*-KO experiments. *ApoE*-KO and C57BL/6 mice were obtained from Vital River Lab Animal Technology Co. Ltd.

Based on previous experience in our laboratory, which involved surgical procedures and late mortality as well as inherent heterogeneity among the CKD models themselves, we initially planned to include 25 animals in each experimental group to ensure the statistical significance and robustness of the findings. The inclusion and exclusion criteria for enrollment and endpoints were predefined. All surviving mice that received treatment were included in the study. Mice that did not survive until the end of the study were excluded (Supplemental Table 6).

The animals were kept in polycarbonate cages in rooms with controlled temperature and humidity, on a 12-hour-light/dark cycle. They were provided with standard chow and tap water ad libitum.

Aortic artery samples from *db/db* and *ApoE*-KO mice administered STZ were provided by Qiming Deng (State Key Laboratory for Innovation and Transformation of Luobing Theory; Key Laboratory of Cardiovascular Remodeling and Function Research of MOE, NHC, CAMS and Shandong Province; Department of Cardiology, Qilu Hospital of Shandong University, Jinan, China) and Ranran Qin (Department of Cardiology, The Affiliated Cardiovascular Hospital of Qingdao University, Qingdao, Shandong 266071, China). The experimental procedure is illustrated in Supplemental Figure 11.

Unilateral ureteral obstruction CKD and the 5/6 nephrotomy procedure were performed as previously described (41). Eight-week-old C57BL/6 mice underwent 7 days of right ureteral obstruction with a nontraumatic titanium microvascular clip for 7 days, followed by the release of this obstruction. After 7 days of recovery, reversal of the obstruction was confirmed by the resolution of hydronephrosis, and the contralateral left kidney was ligated and excised. The sham mice underwent surgical manipulation without ureteral obstruction or nephrectomy. In the 5/6 nephrotomy, the right kidneys of 8-week-old C57BL/6 mice were exposed through a flank incision and removed, and the stump was ligated with a 4-0 silk wire. One week later, the left kidney was exposed, and the upper and lower poles were resected through a lateral incision. All mice were subjected to euthanasia by overdose with phenobarbital (≥150 mg/kg, i.p.) at 15 weeks of age.

For CKD after AKI, a 2-step procedure was used as previously described (42). The operation was performed under isoflurane anesthesia (1.5%–2%). In summary, the right kidney was subjected to cortical electrocautery via a 2 cm incision on the flank, followed by left total nephrectomy through a similar incision after a period of 2 weeks. Control animals underwent sham operations, which involved the removal of the capsule from both kidneys. For metabolic CKD, nephrectomy was performed in the right kidney of 6-week-old ApoE-KO mice. The mice were provided a high-fat diet (20% fat, 20% sugar, and 1.25% cholesterol) for a duration of 6 weeks. When the mice reached 12 weeks of age, they were injected once with low-dose STZ (75–80 mg/kg, i.p.). The model mice exhibited hyperglycemia, IR, and glucose intolerance, as previously described (23, 43). Littermate WT mice underwent a similar sham operation and were injected once with the same volume of saline for 12 weeks. The sham mice were provided a standard laboratory diet with normal Pi (0.5%), while the CKD mice received a high-Pi (1.5%) diet for 12 weeks. *TRIB3*-KO and control mice were injected subcutaneously with 6 × 10^5^ IU/kg vitamin D_3_ daily for 3 days for the vitamin D_3_–induced vascular calcification model. A micellar solution prepared by mixing ethanol, cremophor, and water was used as the solvent. All mice were subjected to euthanasia by overdose with phenobarbital (≥150 mg/kg, i.p.) at 24 weeks of age.

### Blood pressure and PWV measurements.

At the end of the experiment, the mice were anesthetized with 1%–2% isoflurane, and a Millar Mikro-tip 1.4F pressure transducer (SPR-839, Millar Instruments) was inserted into the right carotid artery to measure arterial blood pressure. Ultrasound detectors (Vevo 2100, MX400, EKV imaging) focused on the abdominal aorta or femoral artery to measure the pulse wave propagation distance. The PWV was determined by dividing the distance by the time interval between the pressure wavefronts (44).

### Renal function parameters.

Serum creatinine and blood urea nitrogen levels were measured spectrophotometrically (C011-2-1 and C013-1-1, Nanjing Jiancheng Bioengineering Institute) according to the manufacturer’s instructions (44).

### Human arteries samples.

Human coronary arteries were obtained from cadavers. Carotid plaque samples were obtained via endarterectomy. The source of the samples and patient characteristics have been thoroughly described previously (45). Samples of renal arteries were obtained from receptors and donors in renal transplantation. The clinical characteristics of the serum donors are listed in Supplemental Tables 4–6.

### Cell culture.

Primary human aortic smooth muscle cells (hVSMCs) were commercially obtained from ScienCell Research Laboratories and maintained in smooth muscle cell medium at 37°C in a 5% CO_2_ environment. Only cells from passages 4–6 were utilized for the experiments. Human HEK293T cells were obtained from KeyGene BioTech and cultured in DMEM supplemented with 10% fetal bovine serum (Gibco).

Primary mVSMCs were isolated from the mouse aorta and cultured in smooth muscle cell medium as described previously (46). Only cells from passages 3–5 were utilized for the experiments.

### Plasmids and RNA interference.

Cells were transfected with 2 μg DNA constitutively encoding Smurf1 in pcDNA3.1 vector or empty vector as control using Lipofectamine 3000 reagent (Invitrogen) according to the manufacturer’s protocol. Following transfection, cells were incubated for 24 hours for fluorescence microscopy, 48 hours for luciferase assay and Western blotting, and 7 days for calcification experiments.

HVSMC and HEK293 cells were transfected with 10 nM shRNA or 10 nM negative control (NC) using Lipofectamine 3000 reagent.

The sequences of shRNAs with higher silencing efficiency were as follows: human TRIB3 sequences, sense 5′-CACCGCATCTTGCTGTGAAGAATAACGAATTATTCTTCACAGCAAGATGC-3′, antisense 5′-AAAAGCATCTTGCTGTGAAGAATAATTCGTTATTCTTCACAGCAAGATGC-3′; human ATF4 sequences, sense 5′-CCGGCATGATCCCTCAGTGCATAAACTCGAGTTTATGCACTGAGGGATCATGTTTTTG-3′, antisense 5′-AATTCAAAAACATGATCCCTCAGTGCATAAACTCGAGTTTATGCACTGAGGGATCATG-3′; human CHOP sequences, sense 5′-CCGGCTGCACCAAGCATGAACAATTCTCGAGAATTGTTCATGCTTGGTGCAGTTTTTG-3′, antisense 5′-AATTCAAAAACTGCACCAAGCATGAACAATTCTCGAGAATTGTTCATGCTTGGTGCAG-3′; mouse ATF4 sequences, sense 5′-CACCGCTGCTTACATTACTCTAATCCGAAGATTAGAGTAATGTAAGCAGC-3′, antisense 5′-AAAAGCTGCTTACATTACTCTAATCTTCGGATTAGAGTAATGTAAGCAGC-3′; and mouse CHOP sequences, sense 5′-CACCGCTCTCCAGATTCCAGTCAGACGAATCTGACTGGAATCTGGAGAGC-3′, antisense 5′-AAAAGCTCTCCAGATTCCAGTCAGATTCGTCTGACTGGAATCTGGAGAGC-3′.

### In vitro VSMC calcification.

As previously described, VSMC calcification was induced for 1 week in osteogenic medium containing 2.6 mmol/L Pi. Calcification was quantified by alizarin red staining or by measuring the total calcium content in the cell lysates using the MTB method with a Calcium Assay Kit (C004-2-1, Nanjing Jiancheng Bioengineering Institute). After 48 hours of incubation following transfection, silencing efficiency was assessed by reverse transcription quantitative PCR (RT-qPCR). ALPL activity was measured after 7 days of silencing, and calcium deposition was assessed after 14 days of silencing

### Aortic calcification.

Calcium deposits in aortic sections were stained using alizarin red (Sigma-Aldrich) and Von Koss, as described previously (47). ImageJ software (NIH) was used to quantify the percentage of positively stained areas in each section.

### Aortic stiffness measurements ex vivo.

After euthanizing the mice with isoflurane anesthesia and cervical dislocation, the abdominal aorta was harvested. The isolated aorta was promptly transferred to a precold, oxygenated physiological salt solution. Each ring was placed between two stainless steel wires of a Small Vessel Myograph (DMT 610M, Danish Myo Technology). Wall tension was measured by incrementally increasing the distance (stretch length) between the wires in 50 μm steps, and the measurements were recorded for a duration of 2 minutes per aorta after each increment in stretch length (48).

### E3 ubiquitin ligase prediction.

UbiBrowser 2.0 (49) was used to predict and visualize ubiquitin ligase (E3)/deubiquitinase (DUB) substrate interactions. The likelihood ratio was shown by bubble size, and confidence score was presented by color depth in matrix bubble diagram.

### Multiple sequence alignment and ubiquitination sites prediction.

Further identification of a sequence similarity between species for Smurf1 was assessed using multiple sequence alignment derived from 8 orthologous proteins, including human, gorilla, macaque, mouse, rat, cow, pig, and clawed frogs. The ubiquitination sites of Smurf1 were predicted using UbiNet 2.0 (50).

### Human and mouse RNA-Seq and enrichment analysis.

Total RNA from hVSMCs and mVSMCs from NC siRNA and *siTRIB3*-transfected, *TRIB3*-KO, and littermate mice was obtained. After extracting the total RNA, the samples were subjected to agarose gel electrophoresis, Nanodrop quality assessment, and quantification. For mRNA enrichment, oligo (dT) magnetic beads were used (for degraded RNA or prokaryotic samples, rRNA removal kits [Stranded mRNA-seq Lib Prep Kit, ABclonal] were used directly). RNA-Seq libraries were constructed using a kit that involved RNA fragmentation, randomly primed reverse transcription to generate first-strand cDNA, synthesis of second-strand cDNA using dUTP, end repair of double-stranded cDNA with A-tailing, and ligation of the Illumina adapter for sequencing. The final library was amplified using PCR. The constructed libraries were quality checked using an Agilent 2100 Bioanalyzer, and library quantification was performed using qPCR. Sequencing was performed on an Illumina HiSeq 4000 sequencer. Gene- and transcript-level fragments per kilobase of transcript per million mapped reads (FPKM) were calculated using BallGown. Differential expression analysis was conducted separately at the gene and transcript levels to identify genes or transcripts that showed significant differences in expression between the groups. We cross-ranked the 1,610 upregulated and 1,356 downregulated genes using a dataset of 257 genes associated with VC (DISGENET C0342649, https://disgenet.com/search?view=DISEASES&idents=C0342649&source=ALL&tab=GDA.) The differentially expressed genes were subjected to clustering analysis, GO functional enrichment analysis, and pathway enrichment analysis to further explore their biological relevance using KangChen Biotech’s customized Python software.

### Transcription factor binding signature analysis.

Downregulated DEGs were uploaded to KnockTF2.0 (25) for transcription factor enrichment analysis. Fifty-five transcription factors were listed following the threshold of an FDR-adjusted P value of 0.05. The enriched transcription factors overlapped with vascular calcification-associated 257 genes (DISGENET C0342649), which were visualized using Eveen (51).

### RT-qPCR.

The expression of *TRIB3* and osteogenic factors in the aortic tissues and VSMCs was determined using RT-qPCR. Total RNA was extracted using TRIzol (Invitrogen) and reversely transcribed into cDNA. RT-qPCR was performed using specific primers shown in Supplemental Table 7. The 2−ΔΔCt method was used for comparisons. The relative mRNA levels were normalized to those of GAPDH.

### ELISA.

After centrifugation of the thawed human tissue homogenate, the supernatant was collected and the target protein concentration was measured using a commercially available Human TRIB3 ELISA kit (EH2519, FineTest). The total protein concentration, determined by the BCA assay, was used for normalization.

### Reagents and antibodies.

CHX and MG132 were purchased from MCE. Tunicamycin 4PBA and Pi were purchased from Sigma-Aldrich. Protein A/G agarose gels were obtained from Santa Cruz Biotechnology. The antibodies used are shown in Supplemental Table 8.

### Western blot analysis.

Western blot analysis was performed as described previously (46). Total protein were extracted from vascular tissue and from cells by using the RIPA lysis supplemented with a protease inhibitor (P1005, Beyotime). Proteins were run on 10% SDS-PAGE and transferred to 0.22 μm PVDF membrane. Each membrane was blocked by 5% BSA and incubated with primary antibodies overnight at 4°C. Bound antibodies were detected by horseradish peroxidase–conjugated secondary antibody (1:5,000) and visualized by a chemiluminescent reagent (WBKLS0500, Millipore). For detection, a chemiluminescence instrument (GE, Amersham Imager 680) was employed.

### Coimmunoprecipitation and immunoblot analysis.

For immunoblot analysis, cells or tissues were lysed with RIPA. Protein concentrations were quantified using a BCA Protein Assay Kit (Pierce Biotechnology), and the concentrations of different samples were adjusted to equal levels using the extraction reagent. For IP, whole-cell extracts were lysed in IP buffer (150 mM saline, 50 mM Tris-HCl, 1% NP-40, pH 7.8, and mammalian cell–specific protease inhibitor cocktail [Merck]), and proteins were quantified using the BCA assay. After centrifugation for 10 minutes at 13,000*g*, supernatants were collected and incubated with protein A/G Plus–Agarose immunoprecipitation reagent (Santa Cruz Biotechnology) together with 1 μg of the corresponding antibodies for 4°C incubations overnight. The beads were washed, and immunoprecipitated proteins were eluted from beads by 2X loading buffer and then were incubated at 100°C for 5 minutes. Immunoprecipitated proteins were subjected to Western blot analysis.

### Immunofluorescence microscopy.

hVSMCs were transfected with the expression plasmid for *TRIB3*, Smurf1 WT, and Smurf1ΔC2 on glass coverslips. After 48 hours of transfection, cells were fixed with 4% paraformaldehyde, permeabilized with 0.1% Triton X-100, and blocked with 1% BSA for 30 minutes at room temperature. Next, fixed cells were incubated with the indicated primary antibodies overnight at 4°C. Detection was performed by incubating with fluorescent dye–conjugated secondary antibodies. Nuclei were counterstained with DAPI (Sigma-Aldrich). A leica microscope was used for imaging.

### IHC and immunofluorescence.

The slides were subjected to dewaxing and antigen retrieval using citrate buffer (pH 6.0). Subsequently, they were treated with 3% H_2_O_2_, followed by blocking with 5% BSA for 30 minutes at 37°C. The slides were then incubated with primary antibodies overnight at 4°C. NCs consisting of isotype IgG antibodies were included. The next day, horseradish peroxidase–conjugated secondary antibodies or fluorescein-coupling secondary antibodies were applied for 0.5 hours at room temperature in IHC. Color development was performed using a DAB kit (ZSGB-Bio) for IHC. Hematoxylin or DAPI was used to counterstain the nuclei in the IHC or immunofluorescence staining process.

### Ubiquitination assay.

To analyze the ubiquitination of Myc-TRIB3, HA-expressing HA-ubiquitin (WT), HA-ubiquitin (K48), HA-ubiquitin (K63), and Flag-Smurf1(WT) or its mutants were used to transfect HEK293T cells, and then whole-cell extracts were immunoprecipitated with the Myc-specific antibody and analyzed by Western blotting with an anti-HA antibody. For analysis of ubiquitination of Smurf1, VSMCs were stimulated with Pi.

### Luciferase reporter assays.

To create a WT luciferase construct, we inserted a DNA fragment from the mouse *TRIB3* promoter into a pGL3Basic vector (Promega). Mutant constructs specifically targeting the predicted ATF4 and CHOP sites in the *TRIB3* promoter were generated by site-directed mutagenesis (Promega).

For the luciferase assay, a luciferase reporter plasmid was transfected into mVSMCs cultured in 24-well plates using Lipofectamine 3000. To ensure accurate measurements, we cotransfected the pRL-TK plasmid, which carries Renilla luciferase under the control of the thymidine kinase promoter, as an internal control to monitor transfection efficiency.

Following transfection, cells were exposed to 2.6 mM concentration of Pi for 24 hours. Luciferase activity was subsequently assessed using a Dual-Luciferase Assay Kit (Promega).

### ChIP assay.

Human primary VSMCs infected with 2.6 mM Pi underwent the following procedure: initially, they were incubated with 1% formaldehyde for 10 minutes to facilitate the cross-linking of DNA-protein complexes, which were then halted with 0.125 mol/L glycine. The cells were harvested, lysed, and sonicated to generate genomic DNA fragments ranging from 300 to 1,000 bp in length.

The lysates were subsequently exposed to 2 μg anti-ATF4, anti-CHOP, or normal IgG antibody and left to incubate overnight at 4°C. Protein A/G plus-agarose beads (Santa Cruz Biotechnology) saturated with single-stranded salmon sperm DNA were added to the lysates and incubated for 2 hours. The resulting immunocomplexes were washed and eluted. Then, immunocomplexes were incubated with 1 μL DNase-free RNaseA for 30 minutes at 37°C. The cross-linked DNA was released by incubating the immunocomplexes with proteinase K buffer at 65°C. This DNA was further purified using the phenol/chloroform/isoamyl alcohol method and subsequently amplified by PCR using *TRIB3* promoter-specific primers.

### FAIRE-PCR assay.

After cross-linked DNA were released from immunocomplexes with proteinase K, sample was collected by brief centrifugation with a microfuge and resuspended with 200 μL 10 mM Tris-HCl pH 7.4 to a final volume of 300 μL. Then, the sample was vortex for 10 minutes after adding 300 μL phenol/chloroform/isoamyl alcohol. DNA with an open chromatin region in the aqueous layer (top) was transferred and repeatedly washed with phenol/chloroform/isoamyl alcohol. The final DNA product was precipitated with 1:10 volume 3 M sodium acetate (pH 5.2), 2 volumes 95% ethanol, and 1 μL 20 mg/mL glycogen. Then, the DNA was subsequently amplified by PCR with *TRIB3* promoter and 5UTR and 3UTR-specific primers (52).

### Molecular dynamics simulation for protein-protein interaction.

Human Smurf1 (UniProt accession Q9HCE7) and TRIB3 (UniProt accession Q96RU7) structures were predicted using AlphaFold 2 (53). The dynamics simulation was conducted using the Gromacs 2022.3 (54). The simulation was performed under static conditions at 300 K and atmospheric pressure (1 bar). The force field employed was Amber99sb-ildn, and the solvent used was a water molecule (Tip3p water model). The molecular dynamics simulation system was subjected to energy minimization using the steepest descent method. Subsequently, it underwent 100,000 steps of NVT (canonical ensemble) equilibration and 100,000 steps of NPT (isothermal-isobaric ensemble) equilibration with a coupling constant of 0.1 ps and a duration of 100 ps. Finally, a production molecular dynamics simulation was conducted, comprising 5,000,000 steps with a time step of 2 fs, resulting in a cumulative simulation time of 100 ns. After completing the simulation, a *t* test was performed using the built-in software tools to calculate the root mean square deviation, root mean square fluctuation, and protein radius of gyration for each amino acid movement trajectory. Additionally, the free energy (Molecular Mechanics Poisson-Boltzmann Surface Area method), free-energy landscape, and other relevant data were computed.

### SPR.

Recombinant Smurf1 protein was immobilized on a CM5 sensor chip using an Amine Coupling Kit (BR100050, Cytiva). TRIB3 protein was applied at concentrations ranging from 31 nM to 8 μM, resulting in a kinetic affinity constant of 12.8 nM. Protein-protein interactions were analyzed using the Biacore T200 system, and SPR signals were processed and fitted using the kinetics model in Biacore T200 Evaluation Software to calculate the KD value.

### Statistics.

Data are presented as scatter dot plots with the mean ± SEM. The number of independent biological samples (*n*) and experimental repeats is provided in the figures and their corresponding legends. The normality of the data was evaluated using the Shapiro-Wilk test. In cases where the data did not follow a normal distribution, appropriate transformations (such as logarithmic, reciprocal, or square root) were applied to achieve normality, as determined using the Shapiro-Wilk test. Statistical analysis was conducted using 2-tailed Student’s *t* test for normal distribution with homogeneous variances, unpaired 2-tailed Student’s *t* test incorporating Welch’s correction in scenarios of heterogeneity in variances and the Mann-Whitney *U* test for nonnormally distributed data in 2 independent groups. For datasets with more than 2 independent groups and a single factor, 1-way ANOVA followed by Tukey’s test for homoscedastic data or the Games-Howell test for heteroscedastic data was used. Nonnormal datasets were analyzed using the Steel-Dwass method. Data obtained from the time course were analyzed using 2-way repeated measures ANOVA. Two-group comparisons were performed using unpaired 2-tailed *t* tests or *U* tests. Correlations between the groups were evaluated using Pearson’s correlation test. Statistical significance was set at *P* < 0.05. significant. All analyses were conducted using Graphpad Prism 8.0 software.

### Study approval.

The protocol for this clinical study conformed to the ethical guidelines of the 1975 Declaration of Helsinki and was approved by the Research Ethics Committee of Shandong University. All participants signed an informed consent form before participation in the study. Written informed consent was obtained from all participants. The experimental animal protocols complied with the Management Rules of the Chinese Ministry of Health and were approved by the Ethics Committee affiliated with Qilu Hospital of Shandong University.

### Data availability.

Published ATF4 and CHOP ChIP-Seq data are available from the Gene Expression Omnibus (GSE35681). The transcriptome bulk sequence data have been deposited in the China National Center for Bioinformation (55, 56) (HRA009415, https://ngdc.cncb.ac.cn/gsa-human/submit/hra/list). The study was approved by the Qilu Hospital of Shandong University IRB (12073).

## Author contributions

MZ, HW and YL designed research studies; YL, CM, and YS conducted experiments; SH, HS, and YT acquired data; FW, FC, and ZW analyzed data; CL, HG, MT, and FS provided reagents; and YL and CM wrote the manuscript. All authors read and approved the manuscript. The order of the co-first authors were determined based on their equal contribution to the research. The specific order was agreed upon by both authors, considering their respective roles in the research, with no distinction in the significance of their contributions.

## Supplementary Material

Supplemental data

Unedited blot and gel images

Supplemental video 1

Supporting data values

## Version 1 02/11/2025

In-Press Preview

## Version 2 04/01/2025

Electronic publication
