# Supplementary material for: TRIB3 mediates vascular calcification by facilitating self-ubiquitination and dissociation of Smurf1 in chronic kidney disease
Source: J Clin Invest. 2025 Apr 1;135(7):e175972. doi: 10.1172/JCI175972 (PMC11957692; doi:10.1172/JCI175972)
Supplement: Supplemental data [file jci-135-175972-s254.pdf]

SUPPLEMENTARY MATERIALS

TRIB3 induces vascular calcification through facilitating self ubiquitination and dissociation of Smurf1 in chronic renal disease

Yihui Li[1,2], Chang Ma[1], Yanan Sheng[1], Shanying Huang [1], Huaibing Sun[3], Yun Ti[1], Zhihao Wang[4], Feng Wang[5], Fangfang Chen[6], Chen Li [2], Haipeng Guo[2], Mengxiong Tang[7], Fangqiang Song[8], Hao Wang[2], Ming Zhong[1]

[1]State Key Laboratory for Innovation and Transformation of Luobing Theory; Key Laboratory of Cardiovascular Remodeling and Function Research of MOE, NHC, CAMS and Shandong Province; Department of Cardiology, Qilu Hospital of Shandong University, Jinan, China.

[2]Department of Critical Care Medicine, Qilu Hospital; Innovation Research Center for Sepsis and Multiple Organ Injury, Shandong University, Jinan, China.

[3]Department of Organ Transplantation, Qilu Hospital, Shandong University, Jinan, China.

[4]Department of Geriatric Medicine, Qilu Hospital, Shandong University, Jinan, China.

[5]Department of Critical Care Medicine, Shandong Provincial Hospital, Jinan, Shandong, China

[6]Department of Cardiology, Shandong Provincial Qianfoshan Hospital, Jinan, Shandong, China

[7]Department of Emergency, Qilu Hospital, Shandong University, Jinan, China.

[8]Department of Critical Care Medicine, the Affiliated Tengzhou Hospital of Xuzhou Medical University/ Tengzhou Central People's Hospital, Shandong

Authorship note: YL and CM contributed equally to this work and are co-first authors.

Address correspondence to: Ming Zhong, State Key Laboratory for Innovation and Transformation of Luobing Theory; Key Laboratory of Cardiovascular Remodeling and Function Research of MOE, NHC, CAMS and Shandong Province; Department of Cardiology, Qilu Hospital of Shandong University,

Jinan, China. Phone:86.531.82165801; E-mail: zhongming2sdu@163.com. Or Hao Wang, Department of Critical Care Medicine, Qilu Hospital; Innovation Research Center for Sepsis and Multiple Organ Injury, Shandong University, Jinan, China. Phone:86.531.82165060; E-mail: wanghao34@126.com.

Conflict of interest: The authors have declared that no conflict of interest exists.

TABLES

Supplementary Table 1 Sera Donor characteristics of CKD patients and controls used in the present study

No.	Age (y)	Gender	Cr (μM)	Urea (mM)	TG （mM）	TC (mM)	TP (g/L)	ALB (g/L)	Glu (mM)	UA (μM)	Tbil (μM)	Dbil (μM)	Ca (mM)	P (mM)
CTR1	35	M	56	3.21	1.65	4.27	77.8	45.2	9.92	180.4	13.4	5.1	2.42	1.25
CTR2	62	F	72	6.24	2.05	5.72	70.6	47.4	4.42	251.7	11.2	6.9	2.15	1.42
CTR3	42	F	42	3.15	1.84	5.41	65.6	43.9	5.08	293.4	12.8	6.4	2.13	0.95
CKD1	24	M	324	16.7	5.03	1.78	72.1	40	5.89	514.2	13.5	5.0	1.85	2.51
CKD2	72	M	519	14.5	2.82	0.92	67.8	42.4	4.66	351.4	6.9	4.5	2.02	1.57
CKD3	35	M	521	20.4	2.68	0.68	70.1	40.3	4.92	457.9	6.6	3.5	2.09	1.66
CKD4	55	M	224	8.9	6.49	1.83	65.5	34.7	4.6	551.2	12.3	6.3	2.17	1.45
CKD5	46	M	632	18.7	4.55	2.41	63.7	47.8	13.64	354.7	9.2	4.8	1.74	1.82
CKD6	65	F	849	27.8	5.5	1.25	62.4	33.2	5.41	478.2	9.3	6.9	1.75	2.64
CKD7	29	F	1571	32.4	2.98	1.8	47.9	21.6	4.81	591.7	9.0	5.1	1.85	1.64
CKD8	37	F	421	15.4	3.06	1.48	55.6	37.4	9.92	245.5	9.7	5.7	1.75	2.29
CKD9	42	F	357	10.2	8.82	1.32	69.2	45.6	4.42	249.2	13.4	6.0	2.25	1.23

CR, Creatinine. TG, Triglycerides. TC, Total Cholesterol. TP, Total Protein. ALB, Albumin. Glu, glucose. UA, uric acid. Tbil, Total Bilirubin. Dbil, Direct Bilirubin. Ca, Calcium. P, Phosphorus.

Supplementary Table 2 The characteristics of controls and chronic kidney disease patients with carotid endarterectomy used in the present study

Clinical characteristics		CTR (n =10)	CKD (n = 17)	*P*
Age, y		64.50(59.25,70.00)	67(59.50,72.00)	0.727
Female		2(20.00)	6(35.29)	0.666
Body mass index, kg/m^2		27.83(25.26,29.40)	29.55(27.69,31.66)	0.110
Current drinking		6(60)	10(58.82)	1.000
Current smoking		3(30)	10(58.82)	0.236
CKD staging				<0.001
	1	0	6	
	2	0	6	
	3	0	4	
	4	0	1	
	5	0	0	
Comorbidities				
CAD		6(60)	10(58.82)	0.018
Hypertension		6(60)	9(52.94)	1.000
Diabetes		7(70)	9(52.94)	0.448
Primary parathyroid diseases		0(0)	0(0)	
Laboratory examination				
Total Calcium in Serum, mM		2.37 (2.26, 2.52)	2.30 (2.16, 2.51)	0.434
Serum phosphorus, mM		1.25 (1.03, 1.39)	1.45 (1.20, 1.75)	0.059

CKD, chronic kidney disease. CAD, coronary artery disease. Continuous variables are displayed as median (interquartile range) and other values as n (%). CKD staging based on glomerular filtration rate (Kidney Disease: Improving Global Outcomes, KDIGO guidelines). Unpaired 2-tailed Student t test or Welch correction was used for continuous variables with normal distribution. Comparison of the prevalence of gender and comorbid conditions were made using the $\chi 2$ test or Fisher exact tests if necessary.

Supplementary Table 3 The characteristics of controls and chronic kidney disease patients who provided renal artery samples for the present study.

Clinical characteristics		CTR (n =16)	CKD (n = 16)	*P*
Age, y		39.00 (31.50, 44.75)	42.50 (32.00, 52.75)	0.122
Female		1(6.25)	3(18.75)	0.600
Body mass index, kg/m^2		28.32 (25.93, 32.47)	27.62 (27.27, 30.68)	0.879
Current drinking		11(68.75)	5(31.25)	0.034
Current smoking		10(62.5)	10(62.5)	1.000
CKD staging				<0.001
	1	0	0	
	2	0	0	
	3	0	0	
	4	0	0	
	5	0	16	
Comorbidities				
CAD		3(18.75)	12(75.00)	0.001
Hypertension		4(25)	9(56.25)	0.072
Diabetes		4(25)	6(37.5)	0.446
Primary parathyroid diseases		0(0)	0(0)	
Laboratory examination				
Totol Calcium in Serum, mM		2.41 (2.21, 2.54)	2.17 (1.91, 2.30)	0.002
Serum phosphorus, mM		1.13 (1.05, 1.23)	1.51 (1.10, 1.87)	0.003

CKD, chronic kidney disease. CAD, coronary artery disease. Continuous variables are displayed as median (interquartile range) and other values as n (%). CKD staging based on glomerular filtration rate (Kidney Disease: Improving Global Outcomes, KDIGO guidelines). Unpaired 2-tailed Student t test or Welch correction was used for continuous variables with normal distribution. Comparison of the prevalence of gender and comorbid conditions were made using the $\chi 2$ test or Fisher exact tests if necessary.

Supplementary Table 4 The characteristics of controls and chronic kidney disease patients who provided coronary artery samples for the present study.

Clinical characteristics		CTR (n =10)	CKD (n = 15)	*P*
Age, y		65.50(59.00,69.25)	67(59.00,71.00)	0.836
Female		4(40)	5(33.33)	1.000
Body mass index, kg/m^2		28.61(25.60,32.09)	29.06(25.86,31.25)	0.701
Current drinking		4(40)	6(40)	1.000
Current smoking		5(50)	9(60)	0.697
CKD staging				<0.001
	1	0	1	
	2	0	4	
	3	0	4	
	4	0	5	
	5	0	1	
Comorbidities				
CAD		3(30)	11(73.3)	0.049
Hypertension		3(30)	10(66.67)	0.111
Diabetes		3(30)	8(53.33)	0.414
Primary parathyroid diseases		0(0)	0(0)	
Laboratory examination				
Total Calcium in Serum, mM		2.13(2.11,2.14)	2.07(2.00,2.13)	0.011
Serum phosphorus, mM		1.16(0.91,1.39)	1.62(1.42,1.77)	0.004

CKD, chronic kidney disease. CAD, coronary artery disease. Continuous variables are displayed as median (interquartile range) and other values as n (%). CKD staging based on glomerular filtration rate (Kidney Disease: Improving Global Outcomes, KDIGO guidelines). Unpaired 2-tailed Student t test or Welch correction was used for continuous variables with normal distribution. Comparison of the prevalence of gender and comorbid conditions were made using the $\chi 2$ test or Fisher exact tests if necessary.

Supplementary Table 5 Animals Physiological parameters and renal function in the final

Parameters	Sham		CKD	
	WT	*TRIB3*	WT	*TRIB3*
BW (g)	30.18 ± 3.78	31.09 ± 2.25	22.86 ± 4.83**	22.7 ± 4.78**
SBP (mmHg)	123.64 ± 23.2	116.25 ± 26.26	147.8 ± 34.4	140.36 ± 37.73
DBP (mmHg)	78.23 ± 15.12	77.54 ± 14.45	93.51 ± 23.52	92.42 ± 19.8
Heart rate (bpm)	582.97 ± 133.56	519.93 ± 136.98	585.72 ± 118.91	585.35 ± 119.5
BUN (mM)	7.64 ± 2.03	7.1 ± 1.52	17.18 ± 3.07***	16.9 ± 4.14***
Cr (μM)	13.66 ± 3.47	14.07 ± 3.25	60.2 ± 11.83***	58.55 ± 13.93***
Serum Calcium (mM)	0.151 ± 0.019	0.143 ± 0.013	0.125 ± 0.019**	0.122 ± 0.01**
Serum Phosphate (mM)	2.07 ± 0.17	2.26 ± 0.29	3.61 ± 0.47***	3.39 ± 0.58***

Arithmetic means ± SEM. ** p<0.01, *** p<0.001 statistically significant vs. Sham WT mice. BW, body weight; SBP, systolic blood pressure; DBP, diastolic blood pressure; Bpm, bit per minute; BUN, blood urea nitrogen; Cr, blood creatinine. n=10-12.

Parameters	Sham		DM CKD	
	ApoE KO	*ApoE/TRIB3* KO	*ApoE* KO	*ApoE/TRIB3* KO
BW (g)	29.78 ± 3.93	27.79 ± 5.36	22.03 ± 6**	21.56 ± 6.2**
SBP (mmHg)	112.41 ± 25.76	113.73 ± 25.29	141.39 ± 35.23	133.04 ± 16.32
DBP (mmHg)	72.36 ± 17.1	73.57 ± 15.31	91.76 ± 26.53	86.33 ± 9.73
Heart rate (bpm)	501.47 ± 119.87	579.68 ± 121.67	561.08 ± 148.34	620.69 ± 127.11
BUN (mM)	6.26 ± 2.02	6.6 ± 1.49	20.05 ± 3.58***	18.91 ± 3.17***
Cr (μM)	15.44 ± 2.26	13.35 ± 3.65	64.17 ± 14.3***	59.39 ± 13.89***
Serum Calcium (mM)	0.156 ± 0.021	0.142 ± 0.02	0.121 ± 0.011***	0.133 ± 0.015*
Serum Phosphate (mM)	2.27 ± 0.26	2.38 ± 0.25	4.13 ± 0.48***	3.65 ± 0.71***

Arithmetic means ± SEM. * p<0.05, ** p<0.01, *** p<0.001 statistically significant vs. Sham WT mice. BW, body weight; SBP, systolic blood pressure; DBP, diastolic blood pressure; Bpm, bit per minute; BUN, blood urea nitrogen; Cr, blood creatinine. n=10-12.

Supplementary Table 6 Animals in the present study

**Experimental**	Group	Species	Gender	Source	Background	**Starting number**	**Final number**	**Excluded number from analysis and reasons**
CKD after AKI	Sham WT	Mouse	Male	Joslin Diabetes Center& Vital River Lab	C57BL/6J	25	25	0
	Sham TRIB3 KO					25	25	0
	CKD WT					25	21	4(Dead at final)
	CKD TRIB3 KO					25	18	1(Dead for operation),4(Dead at final),2(severe cachexia)
DM CKD (Hyperlipidemic diabetes mellitus in ApoE KO mice)	Sham ApoE KO	Mouse	Male	Joslin Diabetes Center& Vital River Lab	C57BL/6J	25	23	2(Dead at final)
	Sham TRIB3 ApoE KO			Joslin Diabetes Center& Vital River Lab		25	22	3(Dead at final)
	DM CKD ApoE KO			Joslin Diabetes Center& Vital River Lab		25	20	2(Dead at final),3(severe cachexia)
	DM CKD TRIB3/ApoE KO			Joslin Diabetes Center& Vital River Lab		25	16	5(Dead at final),3(severe cachexia), 1（Die for hemorrhoea when measure intravascular arteria blood pressure）
CKD after AKI	Sham WT	Mouse	Female	Joslin Diabetes Center& Vital River Lab	C57BL/6J	15	15	0
	Sham TRIB3 KO					15	14	1(Dead for operation)
	CKD WT					15	12	3(Dead at final)
	CKD TRIB3 KO					15	11	4(Dead at final)
CKD after AKI	Sham CTR	Mouse	Male	GemPharmatech	C57BL/6J	15	15	0

	Sham SMC-TRIB3^{KO}					15	15	0
	CKD CTR					15	10	5(Dead at final)
	CKD Sham SMC-TRIB3^{KO}					15	12	3(Dead at final)
CKD after AKI	Sham CTR	Mouse	Male	GemPharmatech	C57BL/6J	15	15	0
	Sham EC-TRIB3^{KO}					15	15	0
	CKD CTR					15	11	3(Dead at final), 1(severe cachexia)
	CKD Sham EC-TRIB3^{KO}					15	10	4(Dead at final)
DM CKD (Diabetes mellitus in C57BL/6J background mice)	Sham WT	Mouse	Male	Joslin Diabetes Center& Vital River Lab	C57BL/6J	20	18	2(Dead at final)
	Sham TRIB3 KO					20	20	0
	CKD WT					20	14	4(Dead at final), 2(severe cachexia)
	CKD TRIB3 KO					20	17	3(Dead at final)
Vitamin D_3-Induced Vascular Calcification Model	Sol WT	Mouse	Male	Joslin Diabetes Center& Vital River Lab	C57BL/6J	20	17	3(Dead at final)
	Sol TRIB3 KO					20	19	1(Dead at final)
	VitD WT					20	12	3(Dead for suspected severe peritonitis), 5(Dead at final)
	VitD TRIB3 KO					20	14	6(Dead at final)

Supplementary Table 7 Primers used in the present study

Species	Gene	Forward (5'->3')	Reverse (5'->3')	Product size (bp)	Purpose
Human	TRIB3	TGGTACCCAGCTCCTCTACG	GACAAAGCGACACAGCTTGA	184	qPCR
Human	GAPDH	GGAGCGAGATCCCTCCAAAAT	GGCTGTTGTCATACTTCTCATGG	197	qPCR
Human	Runx2	TGGTTACTGTCATGGCGGGTA	TCTCAGATCGTTGAACCTTGCTA	101	qPCR
Human	Smad1	AGAGACTTCTTGGGTGGAAACA	ATGGTGACACAGTTACTCGGT	157	qPCR
Human	Osterix	GAGGCAACTGGCTAGGTGG	CTGGATTAAGGGGAGCAAAGTC	132	qPCR
Human	Msx2	ATGGCTTCTCCGTCCAAAGG	CGGCTTCTTGTCGGACATGA	174	qPCR
Human	Klf6	GGCAACAGACCTGCCTAGAG	CTCCCGAGCCAGAATGATTTT	122	qPCR
Human	Twist1	GTCCGCAGTCTTACGAGGAG	GCTTGAGGGTCTGAATCTTGCT	156	qPCR
Human	ALPL	ACTGGTACTCAGACAACGAGAT	ACGTCAATGTCCCTGATGTTATG	97	qPCR
Human	COL1A1	GAGGGCCAAGACGAAGACATC	CAGATCACGTCATCGCACAAC	140	qPCR
Human	BMP2	TTCGGCCTGAAACAGAGACC	CCTGAGTGCCTGCGATACAG	83	qPCR
Human	BGLAP	CACTCCTCGCCCTATTGGC	CCCTCCTGCTTGGACACAAAG	112	qPCR
Mouse	TRIB3 BS	CTAGTGCCAGACCCCAGC	ACAGATGGTGCAATCCCGG	156	ChIP-PCR<br>FAIRE-ChIP-PCR
Mouse	TRIB3 5’BS	GCAGGTGGATCGTTGAGTTC	GAAAGGGTTGCTTTGGAGGG	196	FAIRE-ChIP-PCR
Mouse	TRIB3 3’BS	AGGAGCGAGAGAGTGAGAGT	CTCCAGGACAAGGTACCCAG	245	FAIRE-ChIP -PCR

Supplementary Table 8 Antibodies used in the present study

Antibody target	Supplier	Catalog No.	IP (immunoprecipitation)	Dilution (IHC)	Dilution (IF)	Dilution (WB)
Runx2	Abcam	ab76956				1:1000
Runx2	Abcam	ab192256		1:500		
Smad1	Santa Cruz	sc-7965		1:500		1:2000
αSMA	Abcam	ab66133			1:400	
CHOP	Proteintech	66741-1-ig				1:2000
Ubiquitin	Abcam	ab134953				1:5000
PDI	Proteintech	66422-i-ig				1:10000
TRIB3	Santa Cruz	sc34215		1:1000	1:500	1:1000
ATF4	Santa Cruz	sc-390063				1:1000
Smurf1	Santa Cruz	sc-100616				1:1000
Smurf2	Santa Cruz	sc393848				1:1000
Rabbit IgG	CST	5127				1:5000
Myc	CST	2278	1:50			1:1000
Flag	Millipore	14793	1:50			1:1000
HA	CST	3724				1:1000
GAPDH	Proteintech	60004-1-Ig				1:50000
K48	CST	8081				1:1000
K63	CST	5621				1:1000
GRP78	Proteintech	66574-1-Ig				1:20000
p-PERK	Proteintech	82534-1-RR				1:5000
PERK	Proteintech	68482-1-Ig				1:10000
OPN	Santa Cruz	sc-21742		1:50		
Lamin B1	Proteintech	66095-1-Ig				1:20000
Nephrin	Abcam	ab227806			1:2000	

Figure and figure legends

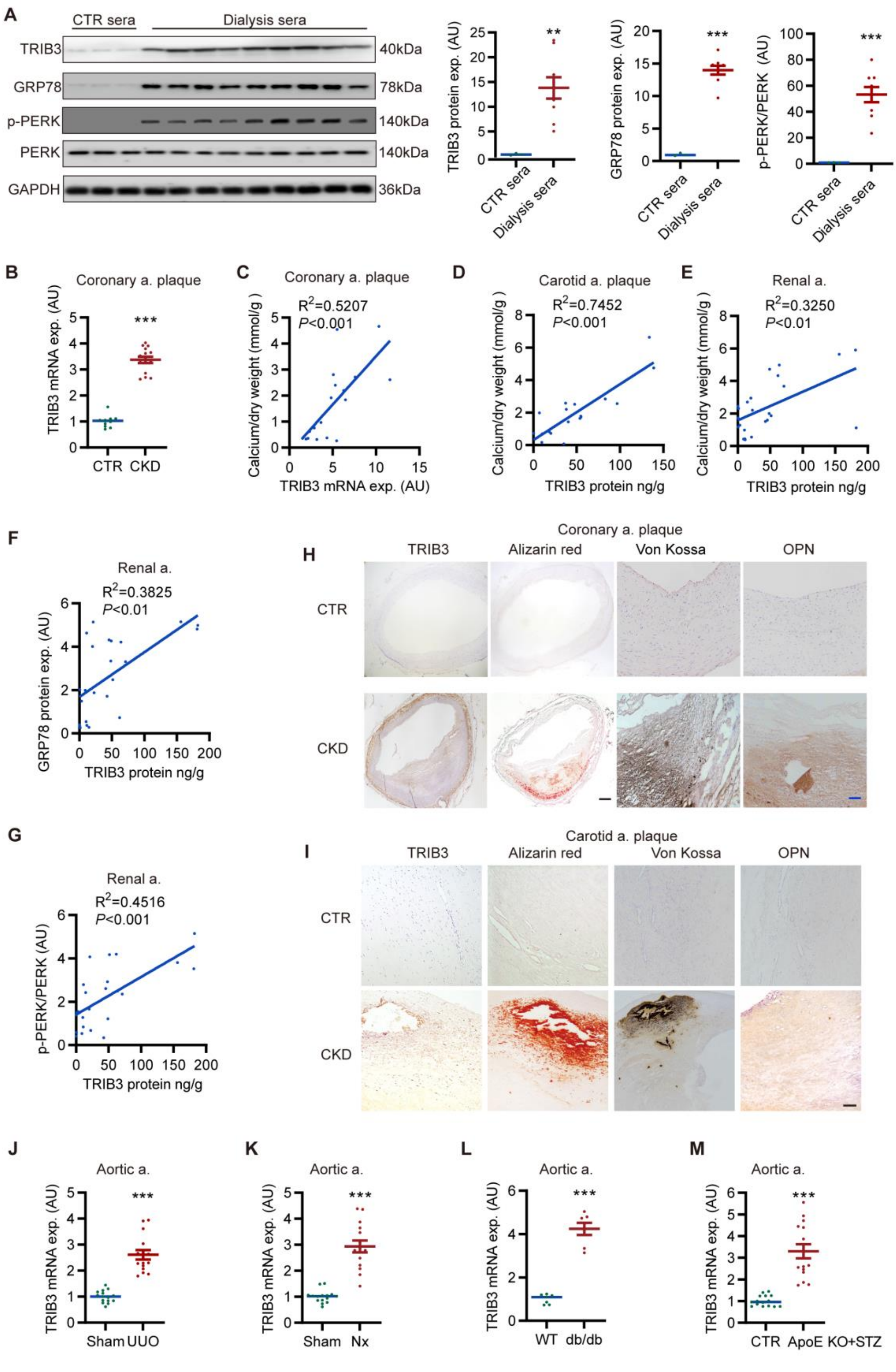

Supplementary Figure 1. Elevated ER stress, TRIB3 expression in CKD environment of hVSMC and coronary artery plaque.

A, Western blots analysis of TRIB3, GPR78 and phosphorylation of PERK expression in hVSMCs following treatment CTR or dialysis patients' sera for 3 days. n=3-9 per group. Statistical analyses were performed using the unpaired 2-tailed Student *t* test with Welch correction. Relative values are compared against those of CTR sera group. **B,** RT-qPCR analysis of TRIB3 relative mRNA expression in CKD patient's coronary artery plaque, n=18 per group. Statistical analyses were performed using the unpaired 2-tailed Student *t* test. Relative values are compared against those of CTR group. **C,** Correlation of TRIB3 relative mRNA expression and calcium deposition in coronary artery plaque from CTR and CKD patients, *P* represents the 2-tailed probability value of the Pearson correlation, n=10-15 per group. **D-E,** Correlation of TRIB3 relative protein expression and calcium deposition in carotid artery plaque and renal artery from CTR and CKD patients, *P* represents the 2-tailed probability value of the Pearson correlation, n=18. **F-G,** Correlation of TRIB3 and GPR78 (D), or TRIB3 and phosphorylation of PERK (E) relative protein expression in renal artery from CTR and CKD patients, *P* represents the 2-tailed probability value of the Pearson correlation, n=24 per group. **H,** Representative original histological images and IHC analysis showing TRIB3 expression and ectopic calcification in coronary artery plaque from control CTR and CKD patients. Alizarin Red staining identifies mid-to-late-stage mineralization, Von Kossa staining identifies late-stage calcification, osteopontin (OPN) serves as a marker for osteogenic differentiation. Black scale bars: 200 μm, blue scale bars: 100 μm. **I**, Representative original histological images and immunohistochemistry (IHC) analysis showing TRIB3 expression and ectopic calcification in carotid plaque from control CTR and CKD patients. Alizarin Red staining identifies mid-to-late-stage mineralization, Von Kossa staining identifies late-stage calcification, osteopontin (OPN) serves as a marker for osteogenic differentiation. Scale bar=100 μm. **J-M,** RT-qPCR analysis of TRIB3 mRNA expression relative to controls in the aortic artery of the

indicated group of mice. Statistical analyses were performed using the unpaired 2-tailed Student *t* test with Welch correction. Relative values are compared against those of Sham, WT or CTR group. Scatter dot plots and arithmetic means ± SEM (arbitrary units (AU)). $^{**}P < 0.01$, $^{***}P < 0.001$ statistically significant *vs.* Sham, WT, CTR, or CTR sera.

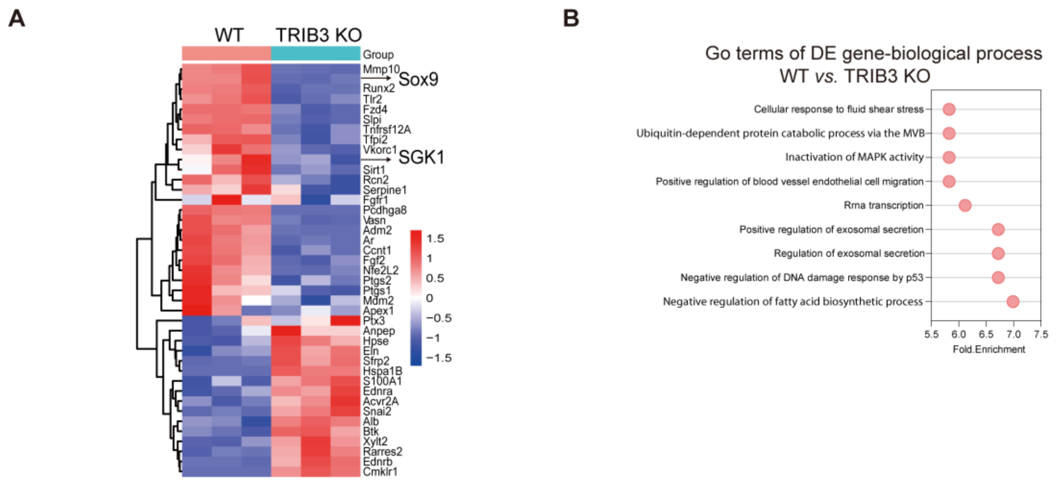

Supplementary Figure 2.

A, Heatmap of DEGs of vascular calcification phenotype gene cluster (DisGenNet C0342649) in mVSMC of WT and TRIB3 knockout mice. **B,** GO analysis of DEGs in mVSMC from WT and TRIB3 knockout mice.

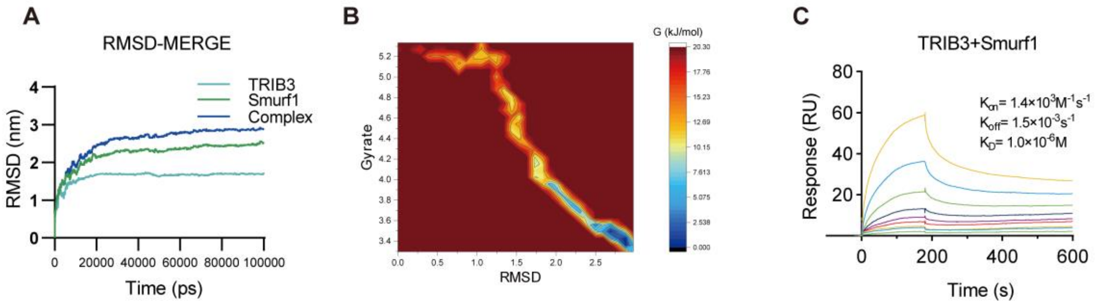

Supplementary Figure 3. Molecular dynamics simulation of human Smurf1 and TRIB3 interaction.

A, Root Mean Square Deviation of human Smurf1, TRIB3 and protein-protein interact complex by Gromacs. **B,** Free Energy Landscape of Smurf1and TRIB3 complex. Human Smurf1 (Q9HCE7) and TRIB3 (Q96RU7) structure was predicted with AlphaFold 2. C, Membrane-binding analysis via surface plasmon resonance (SPR) spectroscopy. SPR sensorgrams having association time intervals of 200 s and dissociation time intervals of 800. RU values were computed relative to the RUL values at each concentration. The presented results represent one of three independent replicates.

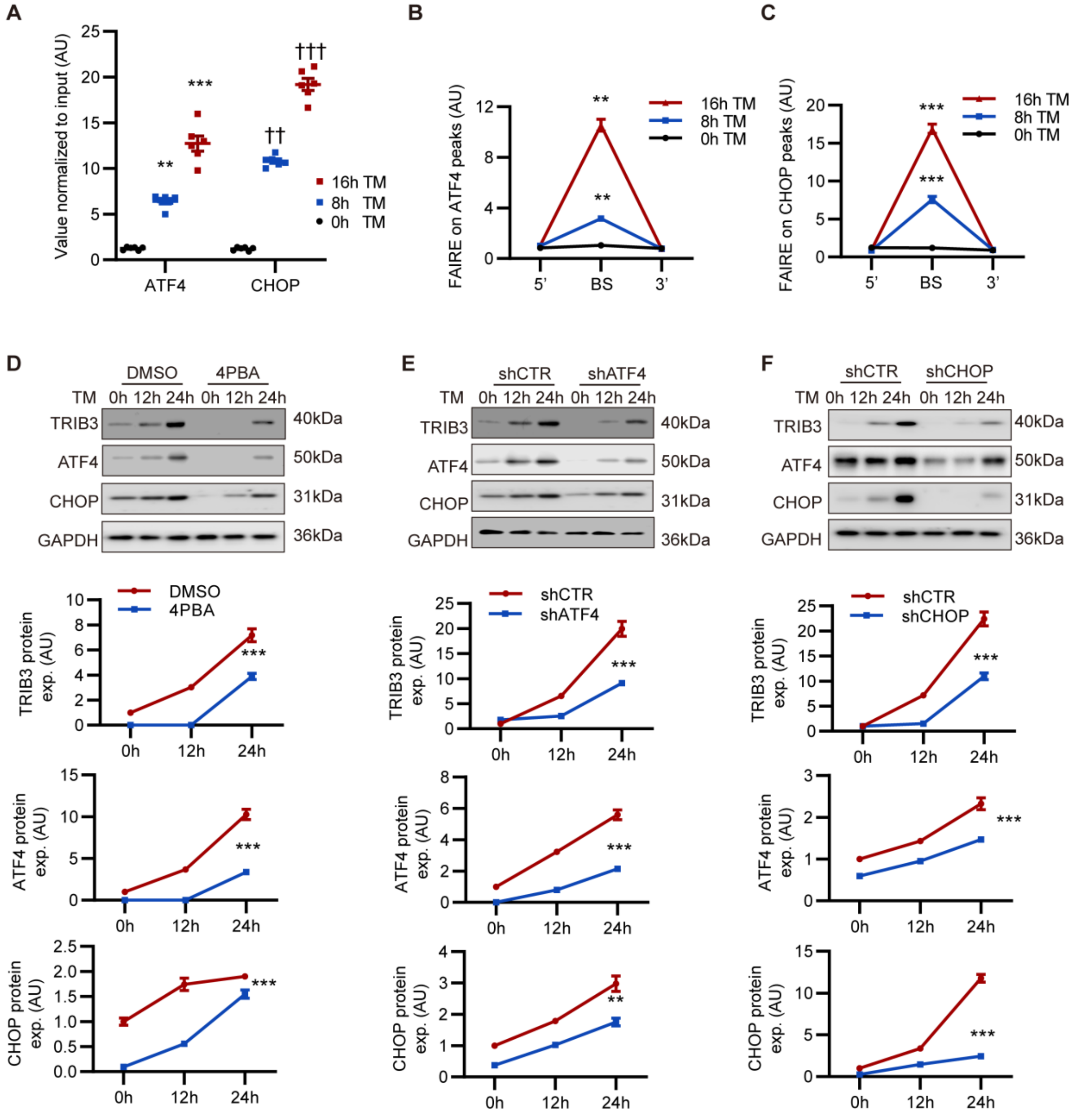

Supplementary Figure 4. ATF4 and CHOP induced TRIB3 transcription in tunicamycin stimuli

A, ChIP-PCR showing enrichment of both ATF4 and CHOP at the TRIB3 binding site (BS) in mVSMC treated 0.1 μg/ml tunicamycin (TM) for 24h. Statistical analyses were performed using the 1-way ANOVA. **$P < 0.01$, ***$P < 0.001$ statistically significant *vs.* 0h TM treatment (ATF4), ††$P < 0.01$, †††$P < 0.001$ statistically significant *vs.* 0h TM treatment (CHOP). **B-C,** FAIRE-ChIP PCR was performed on TM for indicated time in mVSMC. Statistical analyses were performed using the repeated measures two-way ANOVA. **D-F**, Representative western blots of TRIB3, ATF4, and CHOP in mVSMC stimulated with treated with TM at the indicated times after pretreated with 5 μM 4-Phenylbutyric acid (4PBA) for 12h, or transfected with shRNA for knockdown ATF4 and CHOP for

48h. Statistical analyses were performed using the repeated measures two-way ANOVA. **P* < 0.05, ***P* < 0.01, ****P* < 0.001 statistically significant *vs.* 0h treatment, DMSO, or shCTR. Each experiment was repeated independently for six times. Scatter dot plots and arithmetic means ± SEM (AU).

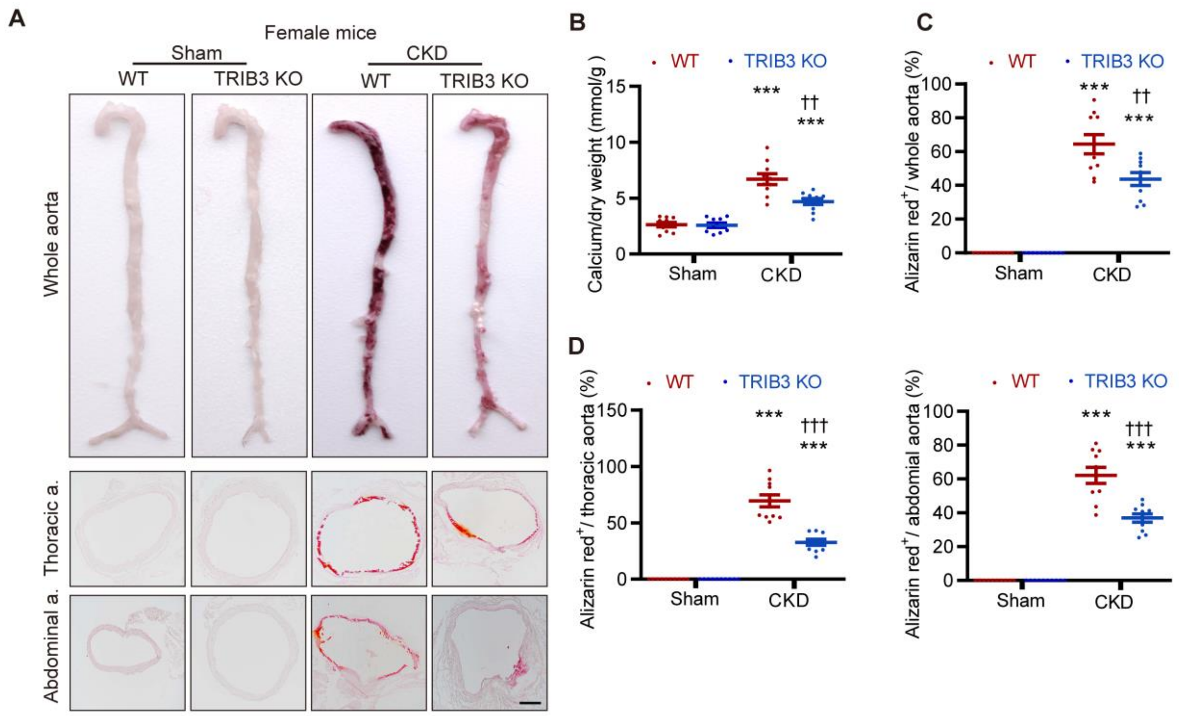

Supplementary Figure 5. Effect of TRIB3 deficiency during AKI-induced CKD vascular calcification in female mice.

A, Representative Alizarin red staining of whole aorta and thoracic and abdominal aorta section images showing aortic alizarin red staining in AKI-induced CKD female mice. Scale bar: 50μm. Calcified areas are shown as red staining. **B,** Calcium content analysis in the aortic arch of AKI-induced CKD female mice, normalized by dry weight. Statistical analyses were performed using the 2-way ANOVA. **C-D,** The ratio of Alizarin Red-positive area to the whole aortic area, thoracic aorta, and abdominal aorta in the indicated group of mice. Statistical analyses were performed using the 2-way ANOVA. Statistical analyses were performed using the 2-way ANOVA. ****P* < 0.001 statistically significant *vs.* Sham WT mice; ††*P* < 0.01, †††*P* < 0.001 statistically significant *vs.* CKD WT mice. n =10 per group. Scatter dot plots and arithmetic means ± SEM (AU).

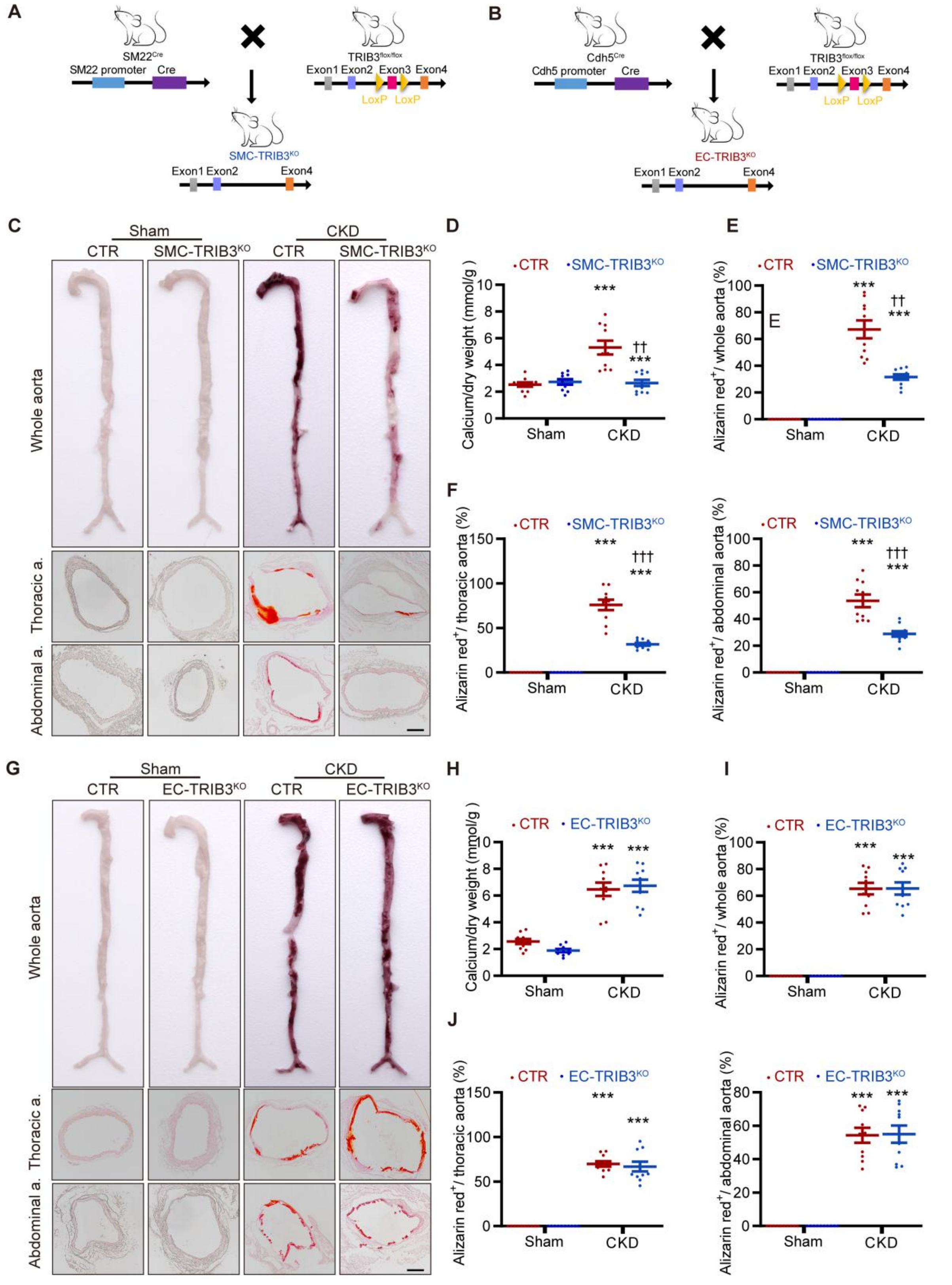

Supplementary Figure 6. **Impact of TRIB3 deficiency in vascular smooth muscle cell- or endothelial cell-specific knockout mice**

A, Schematic diagram of transgenic mice used to generate adult smooth muscle cell-specific TRIB3

knockout mice. **B,** Schematic diagram of transgenic mice used to generate adult vascular endothelial cell -specific TRIB3 knockout mice. **C,** Representative Alizarin red staining of whole aorta and thoracic and abdominal aorta section images showing aortic alizarin red staining in the indicated group of mice. Scale bar: 50μm. Calcified areas are shown as red staining. **D,** Calcium content analysis in the aortic arch of indicated group of mice, normalized by dry weight. Statistical analyses were performed using the 2-way ANOVA. **E-F,** The ratio of Alizarin Red-positive area to the whole aortic area, thoracic aorta, and abdominal aorta in the indicated group of mice. Statistical analyses were performed using the 2-way ANOVA. **G,** Representative Alizarin red staining of whole aorta and thoracic and abdominal aorta section images showing aortic alizarin red staining in the indicated group of mice. Scale bar: 50μm. Calcified areas are shown as red staining. **H,** Calcium content analysis in the aortic arch of indicated group of mice, normalized by dry weight. Statistical analyses were performed using the 2-way ANOVA. **I-J,** The ratio of Alizarin Red-positive area to the whole aortic area, thoracic aorta, and abdominal aorta in the indicated group of mice. Statistical analyses were performed using the 2-way ANOVA. ****P* < 0.001 statistically significant *vs.* Sham CTR mice; ††*P* < 0.01, †††*P* < 0.001 statistically significant *vs.* CKD CTR mice. n =10 per group unless otherwise indicated. Scatter dot plots and arithmetic means ± SEM (AU).

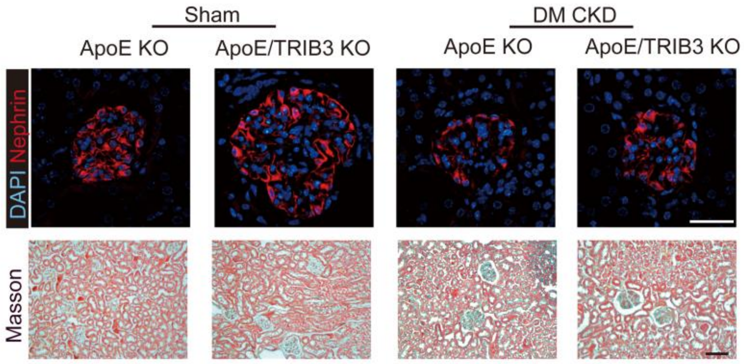

Supplementary Figure 7. The renal injury of metabolic CKD in diabetic ApoE knockout mice. Upper lane, Representative immunofluorescence of Nephrin in kidney tissue in diabetic ApoE knockout mice. The 4',6-diamidino-2-phenylindole (DAPI) indicated the nuclei as blue, Nephrin indicated as red. Scale bars: 20 μm. Lower lane, Representative Masson's trichrome stain for kidney tissue in diabetic *ApoE* knockout mice. The above results are representative figures from six mice.

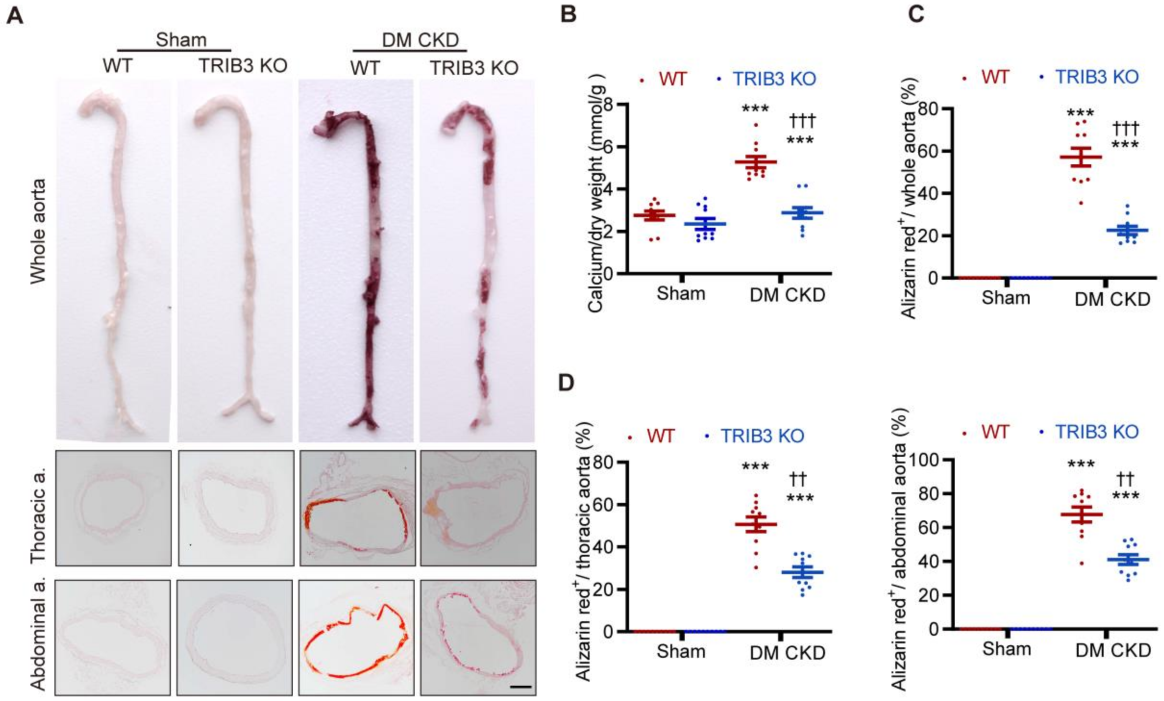

Supplementary Figure 8. **Impact of TRIB3 deficiency in diabetic mice**

A, Representative Alizarin red staining of whole aorta and thoracic and abdominal aorta section images showing aortic alizarin red staining in the indicated group of mice. Scale bar: 50μm. Calcified areas are shown as red staining. **B,** Calcium content analysis in the aortic arch of indicated group of mice, normalized by dry weight. Statistical analyses were performed using the 2-way ANOVA. **C-D,** The ratio of Alizarin Red-positive area to the whole aortic area, thoracic aorta, and abdominal aorta in the indicated group of mice. Statistical analyses were performed using the 2-way ANOVA. ***$P < 0.001$ statistically significant *vs.* Sham CTR mice; ††$P < 0.01$, †††$P < 0.001$ statistically significant *vs.* CKD CTR mice. n=10 per group unless otherwise indicated. Scatter dot plots and arithmetic means ± SEM (AU).

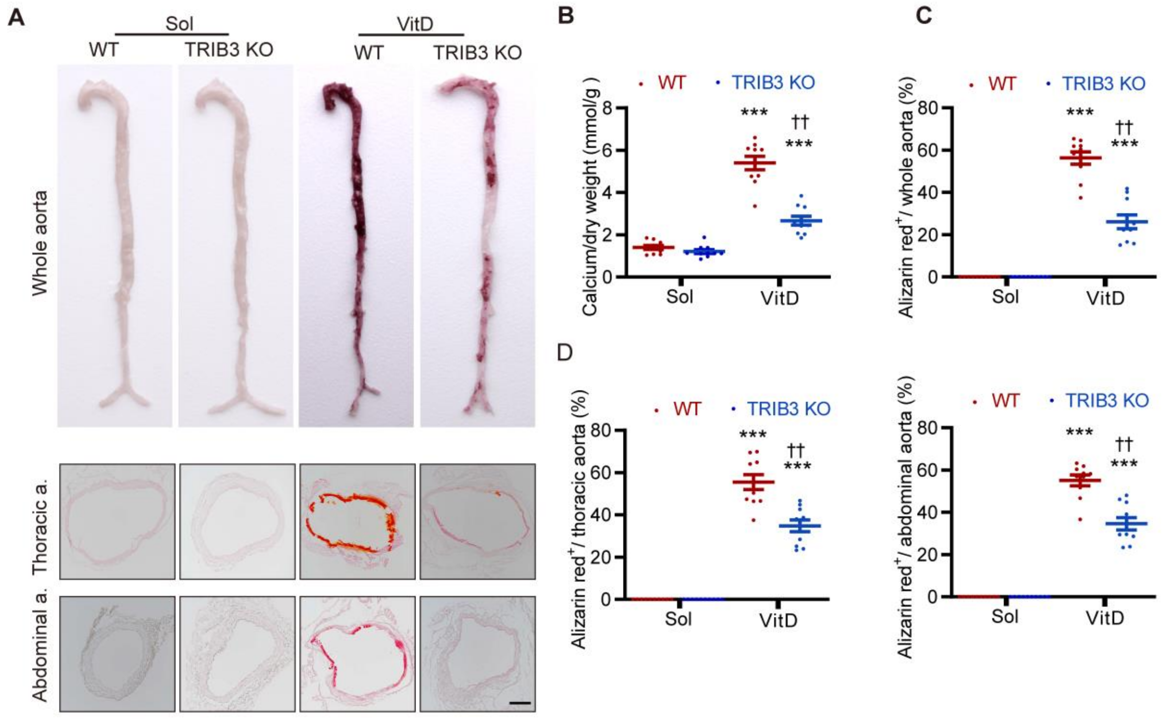

Supplementary Figure 9. **Impact of TRIB3 deficiency in VitD mice**

A, Representative Alizarin red staining of whole aorta and thoracic and abdominal aorta section images showing aortic alizarin red staining in the indicated group of mice. Scale bar: 50μm. Calcified areas are shown as red staining. **B,** Calcium content analysis in the aortic arch of indicated group of mice, normalized by dry weight. Statistical analyses were performed using the 2-way ANOVA. **C-D,** The ratio of Alizarin red-positive area to the whole aortic area, thoracic aorta, and abdominal aorta in the indicated group of mice. Statistical analyses were performed using the 2-way ANOVA. ****P* < 0.001 statistically significant *vs.* Sol WT mice; ††*P* < 0.01 statistically significant *vs.* VitD WT mice. n=10 per group unless otherwise indicated. Scatter dot plots and arithmetic means ± SEM (AU). Sol, Solvent. VitD, vitamin D_3.

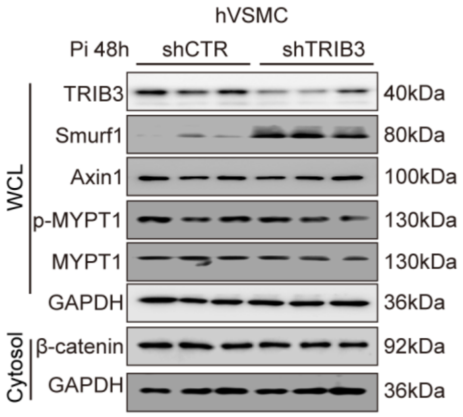

Supplementary Figure 10. Western Blot Analysis of Wnt pathway in Pi-treated hVSMCs

Representative western blots showing the expression of the indicated Wnt pathway proteins in hVSMC treated with Pi (2.6 mM, 48h). The presented results represent one of three independent replicates.

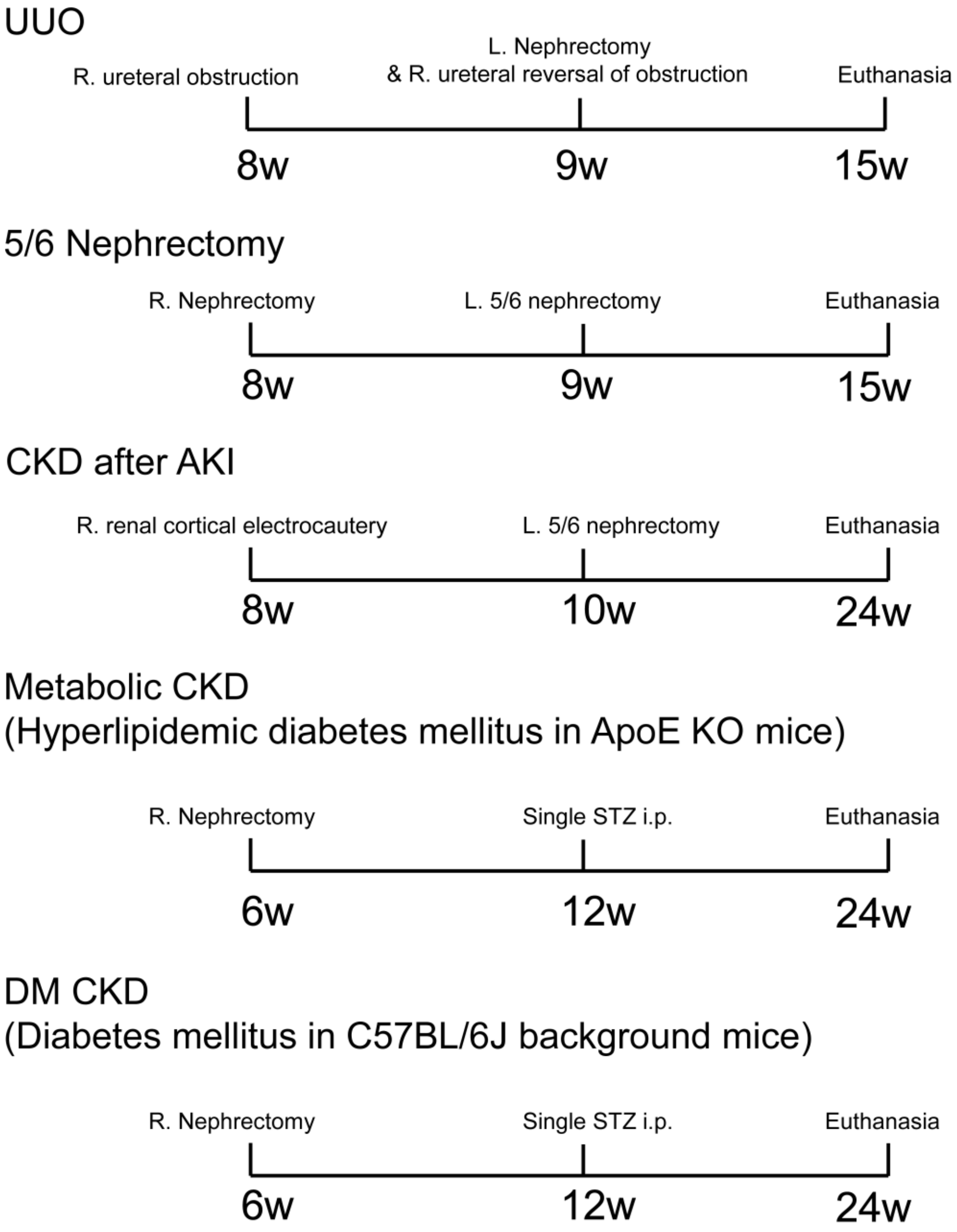

Supplementary Figure 11. Animal experiment flow chart

Right (R.), Left (L.), 5/6 nephrotomy (Nx) mice, unilateral ureteral obstruction (UUO) mice, chronical renal disease (CKD), streptozotocin (STZ), intraperitoneal injection (i.p.).
