# Supplementary material for: TRIB3 mediates vascular calcification by facilitating self-ubiquitination and dissociation of Smurf1 in chronic kidney disease
Source: J Clin Invest. 2025 Apr 1;135(7):e175972. doi: 10.1172/JCI175972 (PMC11957692; doi:10.1172/JCI175972)

Full unedited gel for Figure 1A

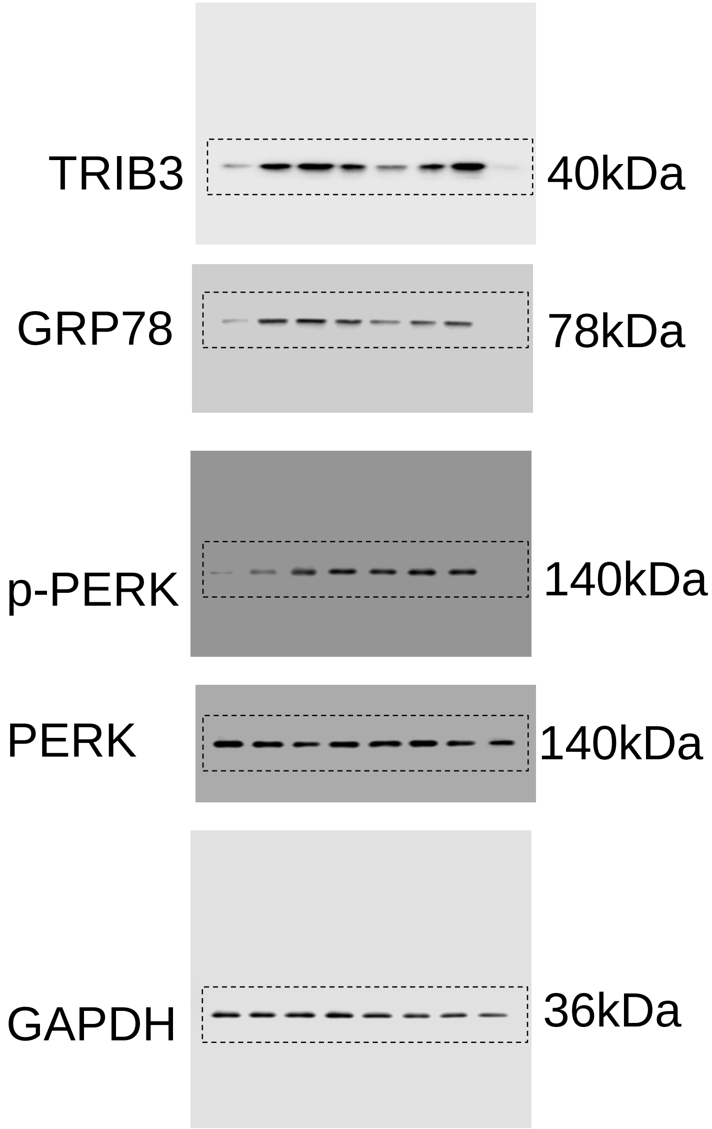

Full unedited gel for Figure 1G

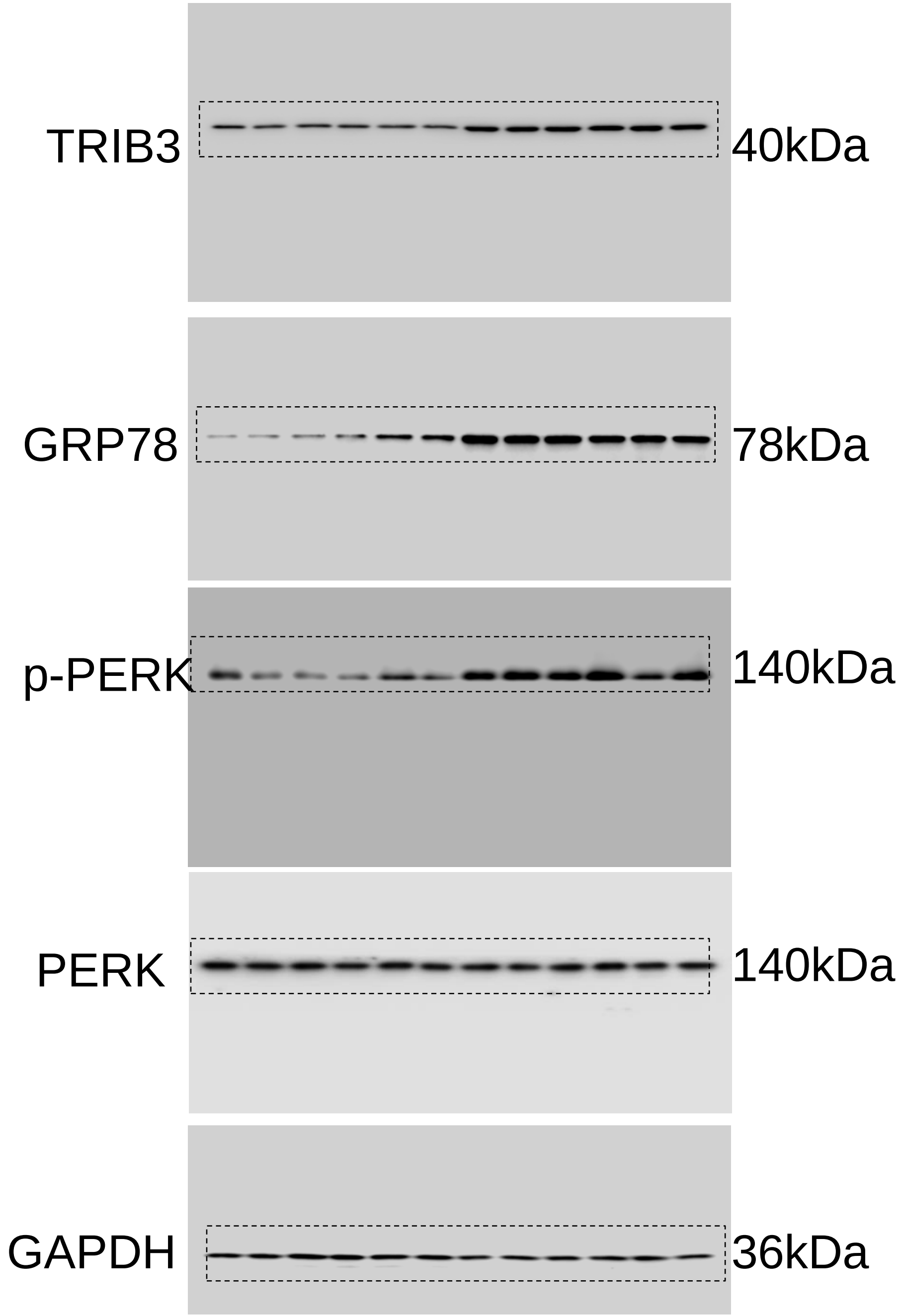

Full unedited gel for Figure 2E

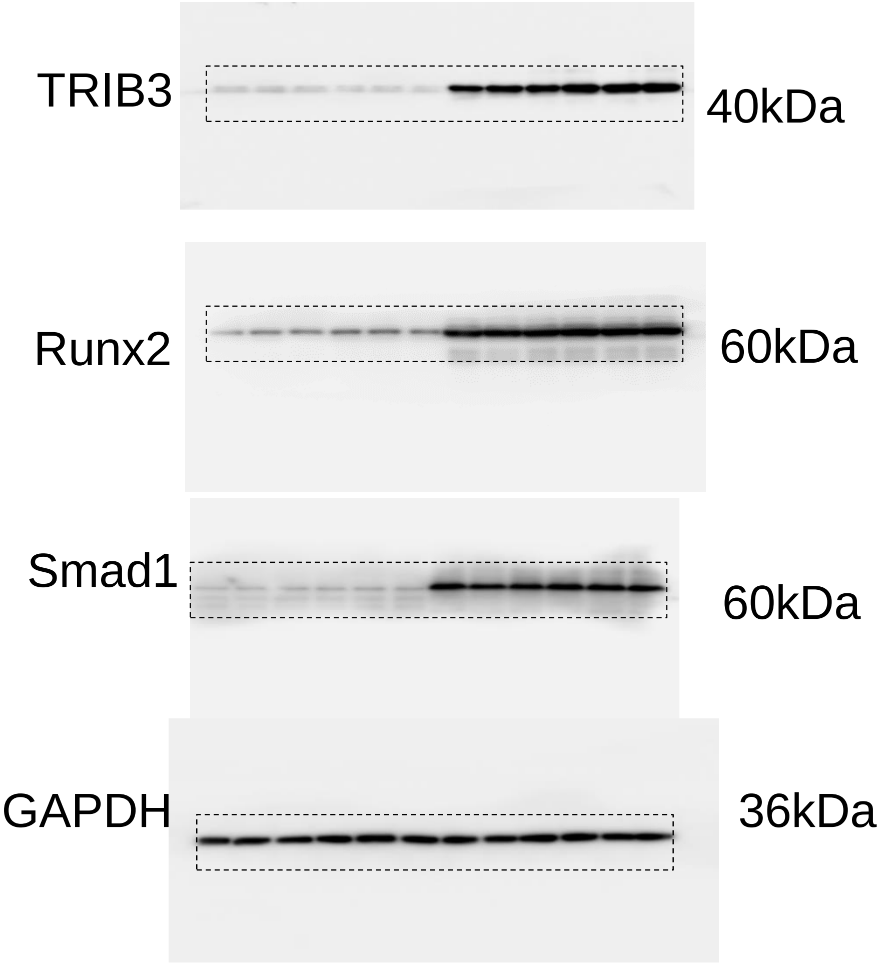

Full unedited gel for Figure 2F

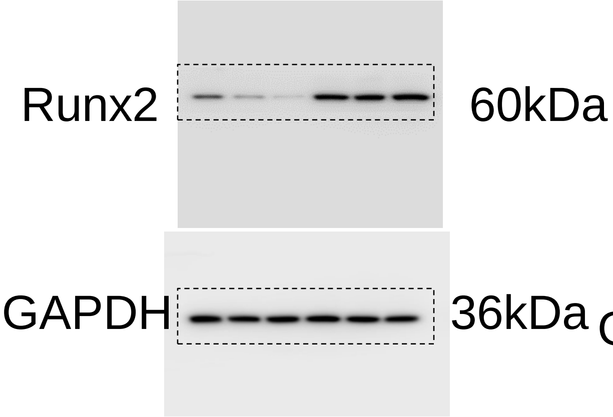

Full unedited gel for Figure 2G

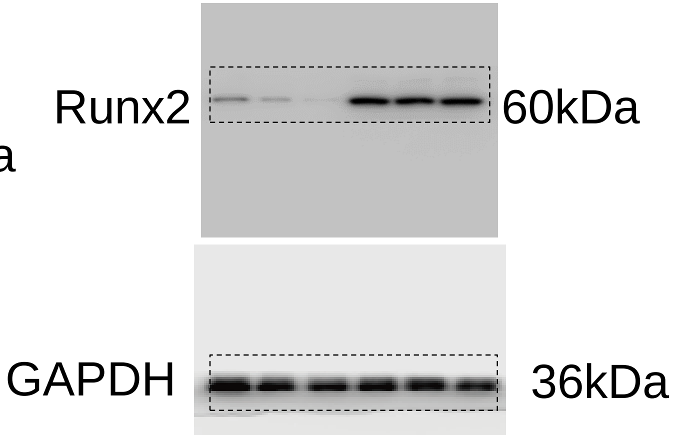

Full unedited gel for Figure 2H

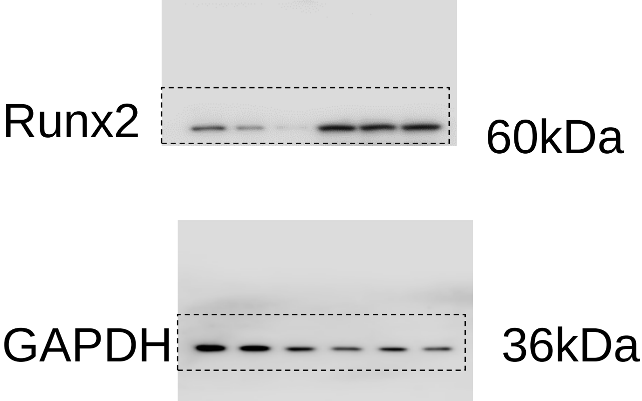

Full unedited gel for Figure 2I

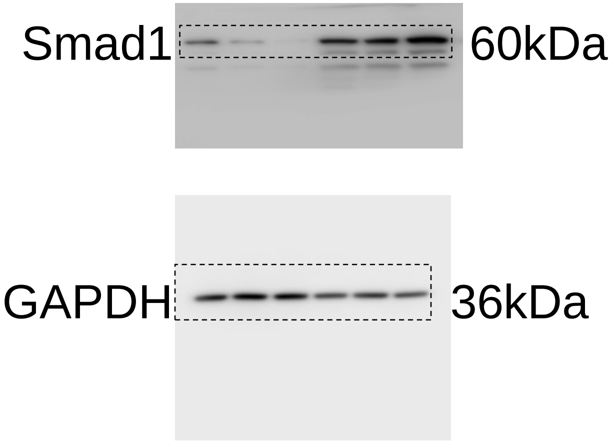

Full unedited gel for Figure 2J

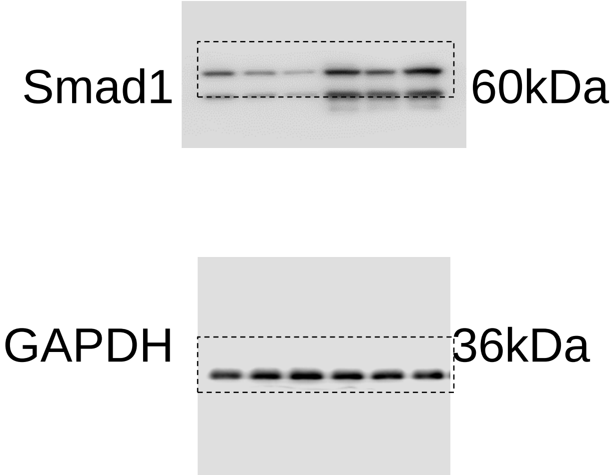

Full unedited gel for Figure 2K

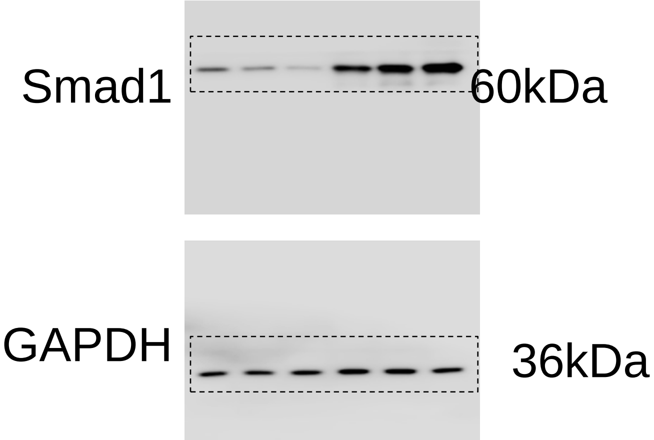

Full unedited gel for Figure 3B

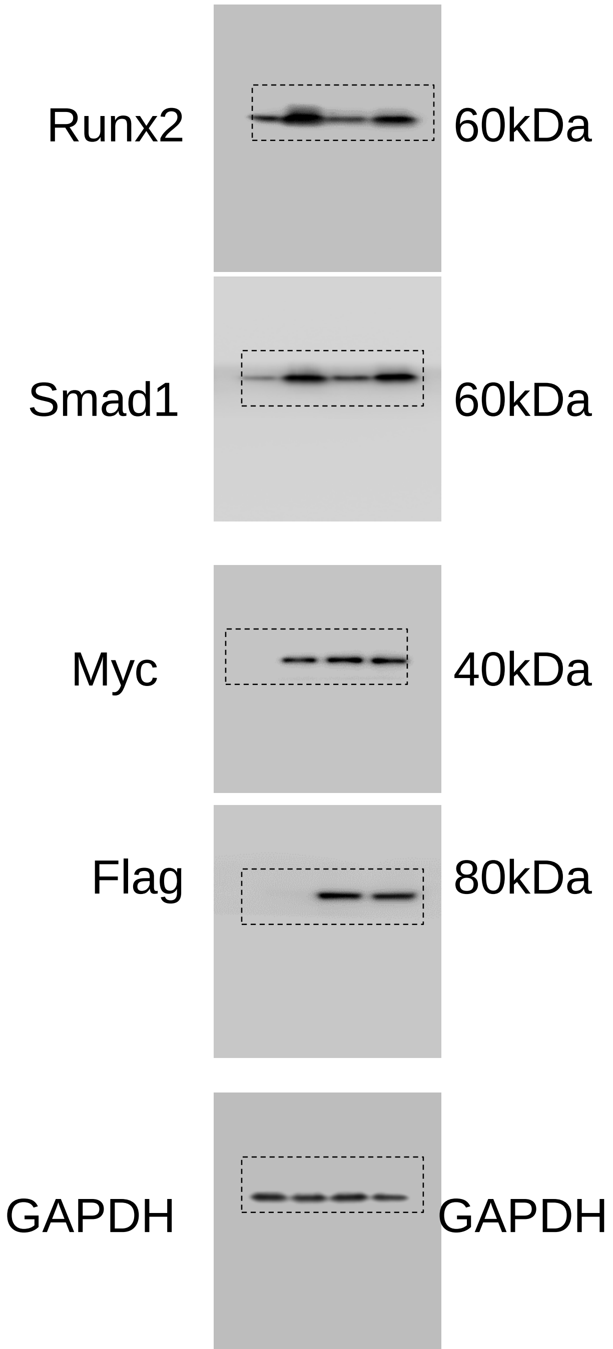

Full unedited gel for Figure 3C

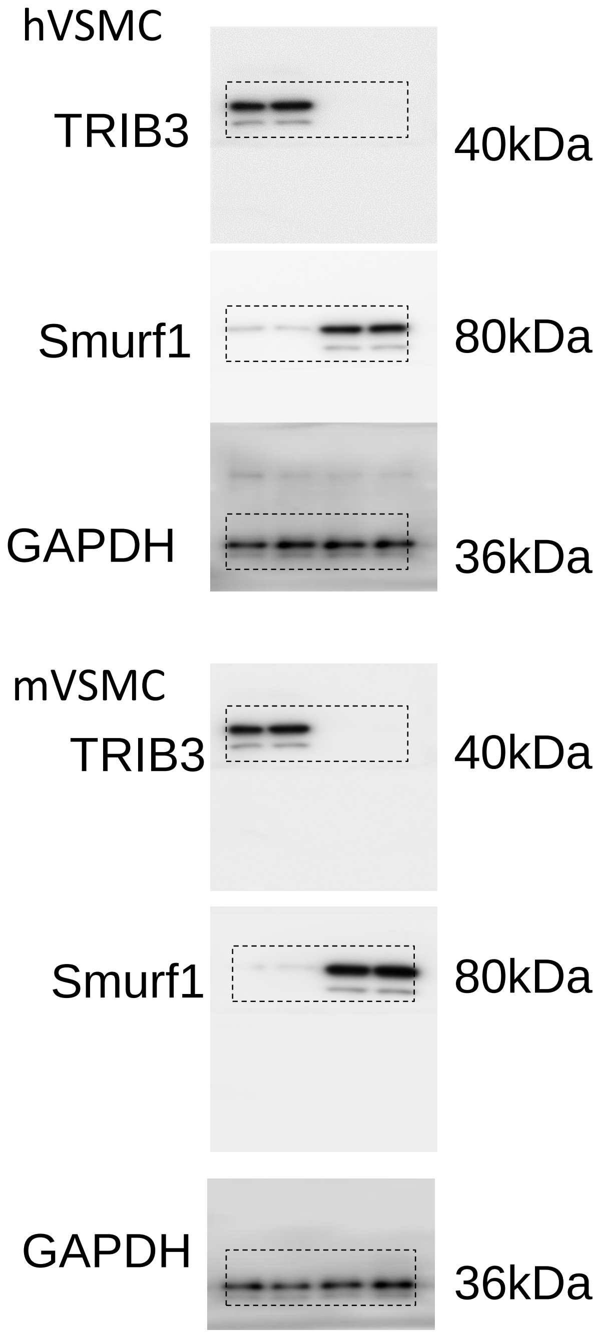

Full unedited gel for Figure 3D

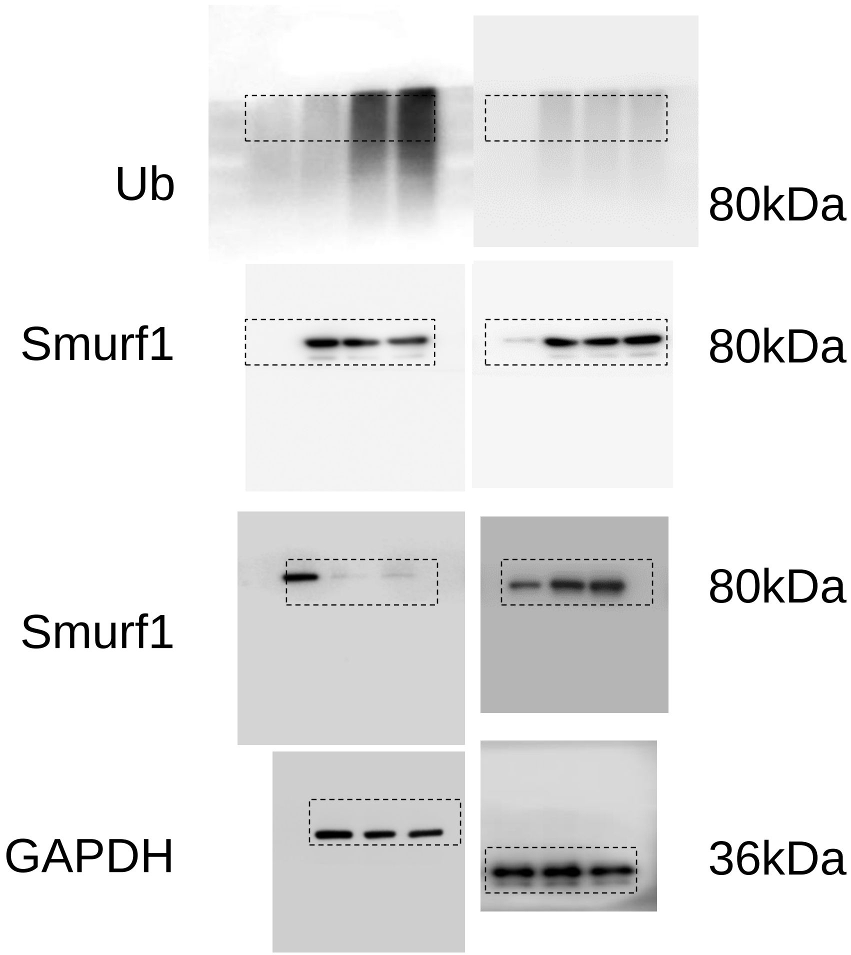

Full unedited gel for Figure 3E

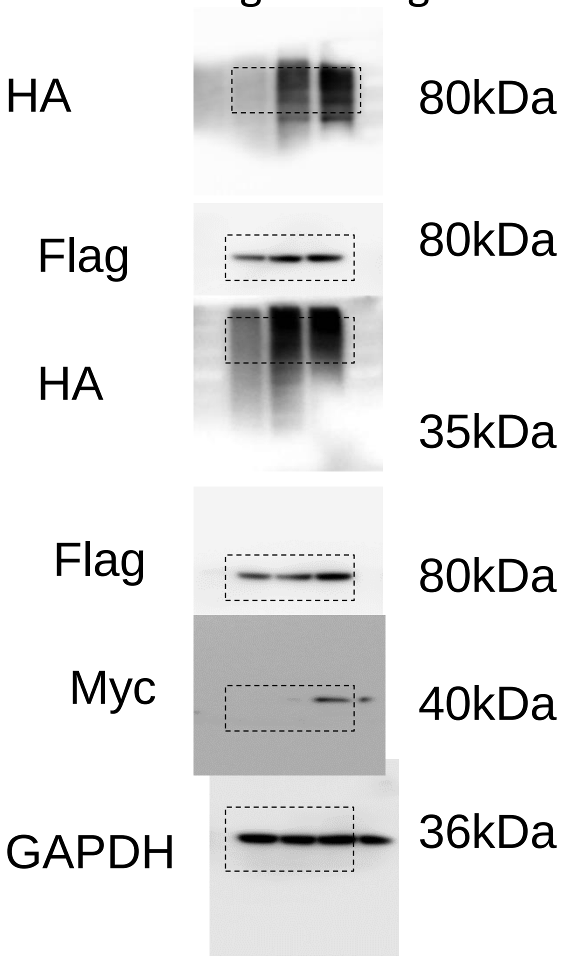

Full unedited gel for Figure 3F

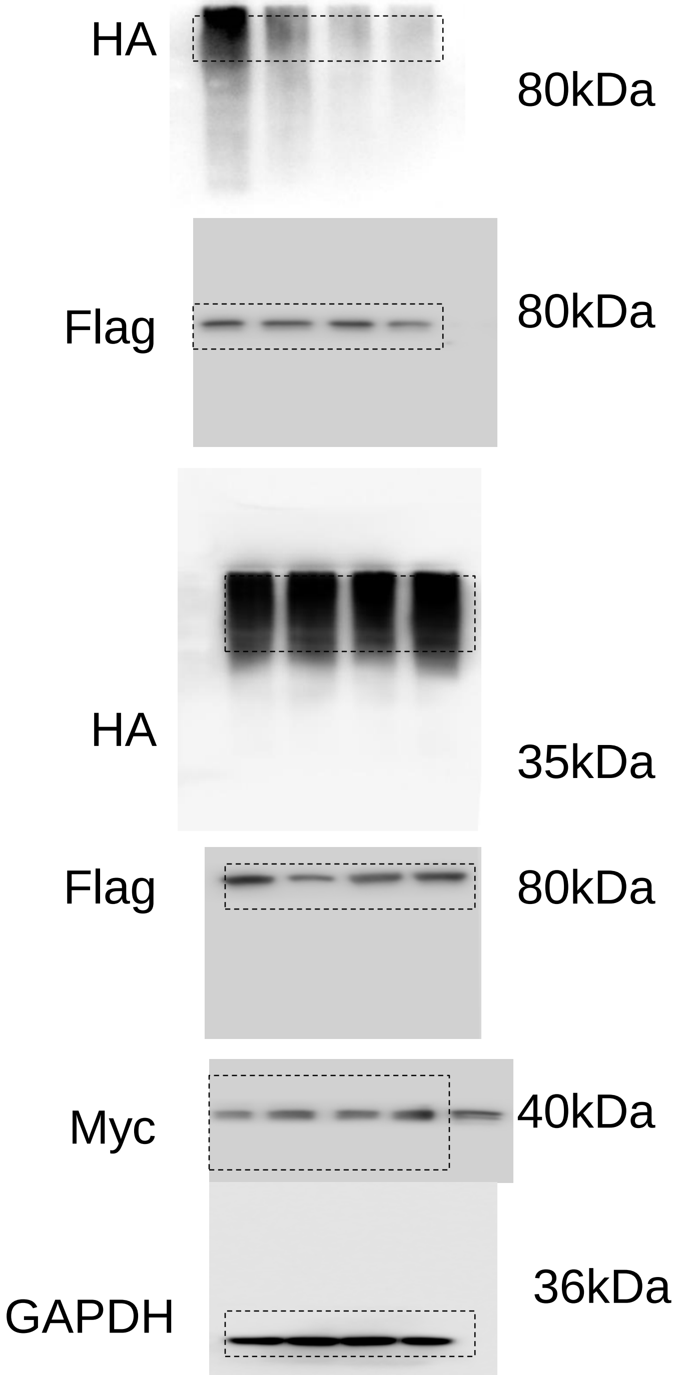

Full unedited gel for Figure 3H

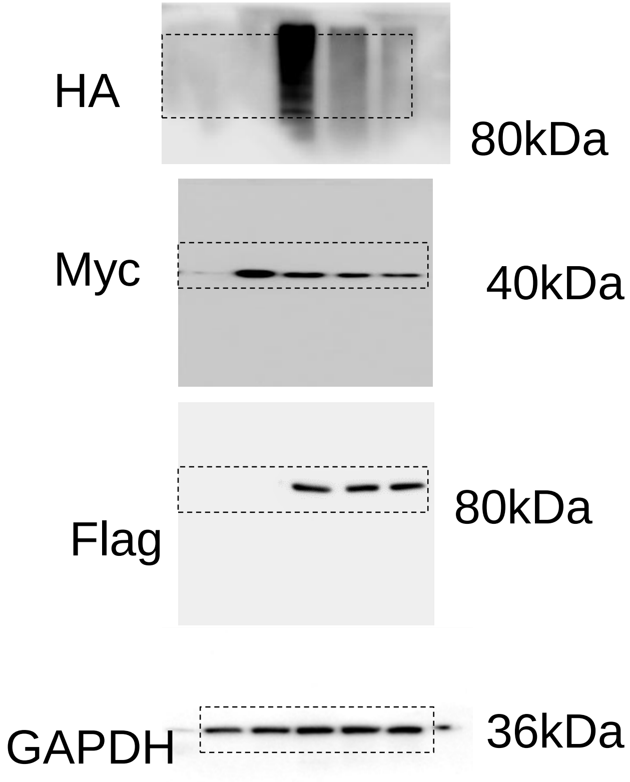

Full unedited gel for Figure 4C

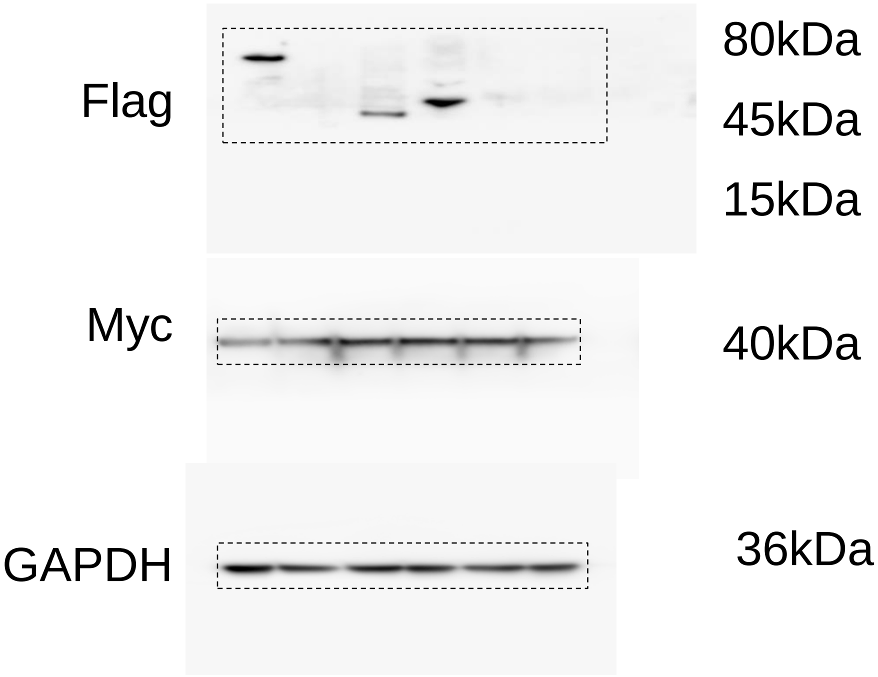

Full unedited gel for Figure 4E

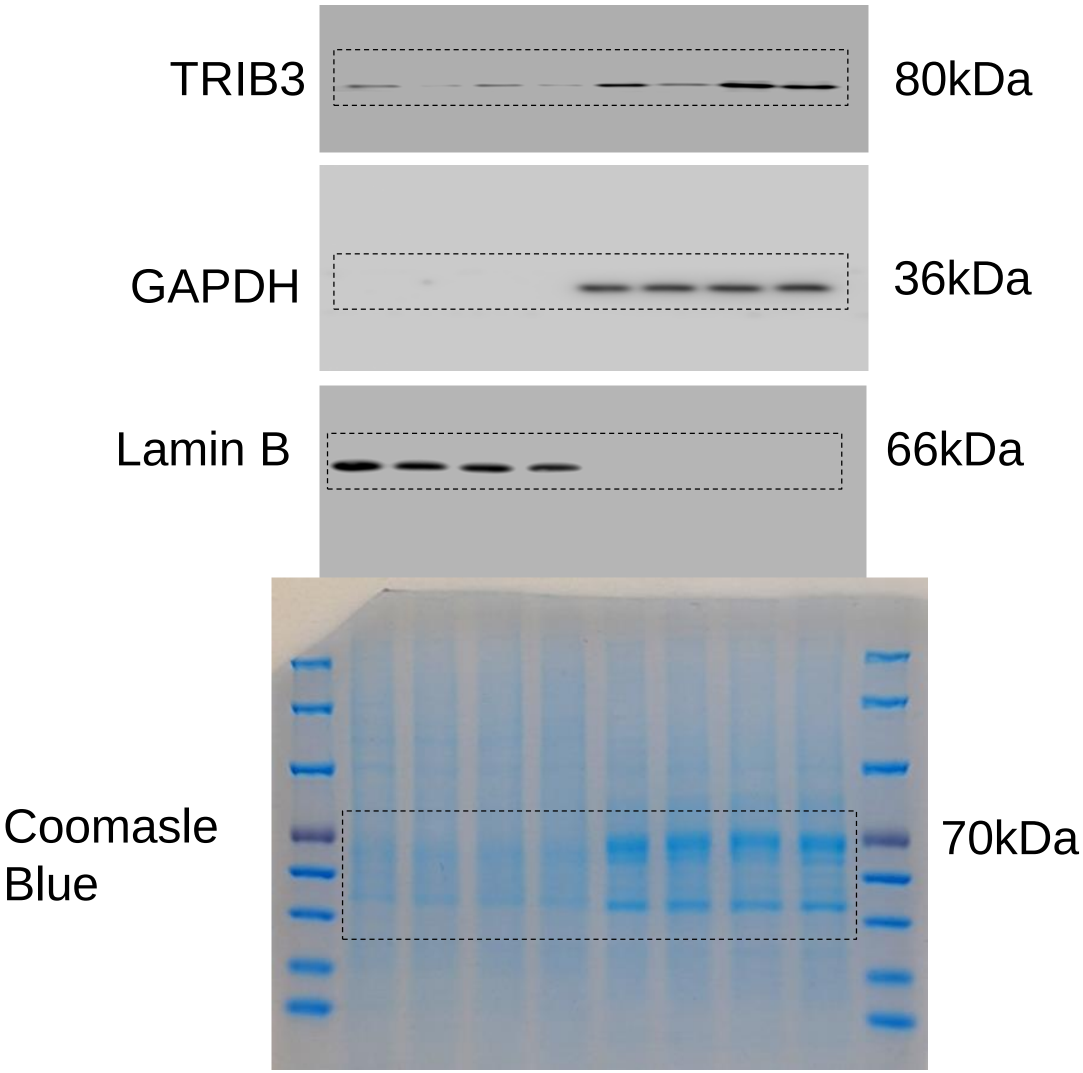

Full unedited gel for Figure 4F

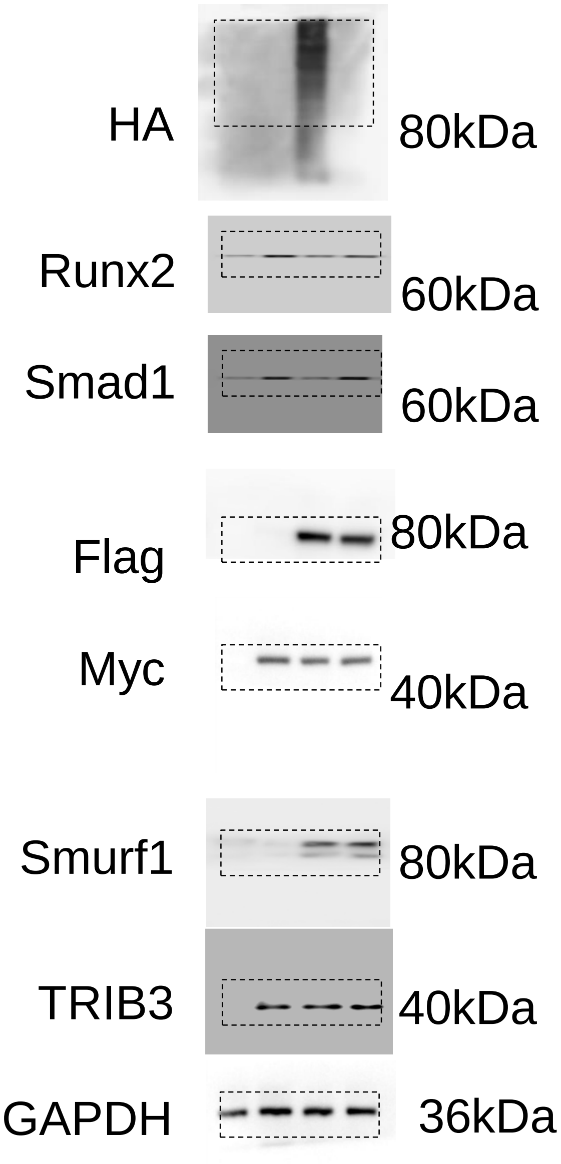

Full unedited gel for Figure 5G

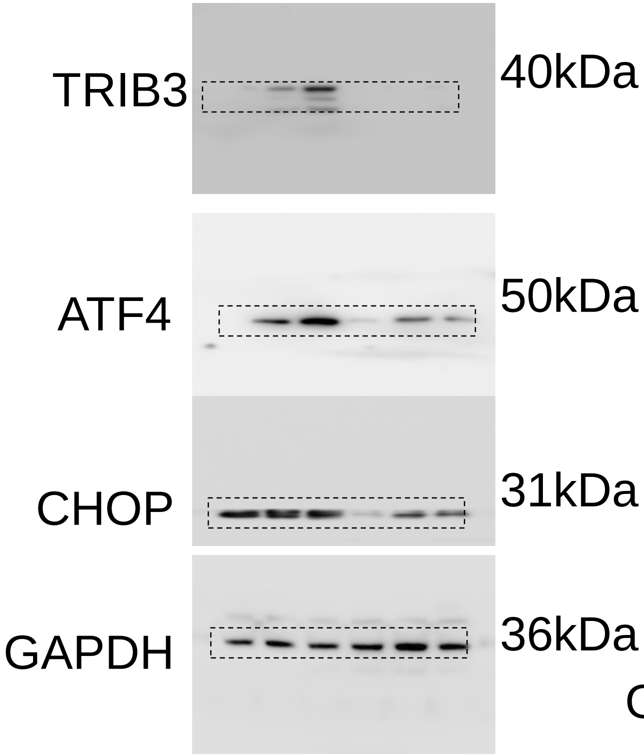

Full unedited gel for Figure 5H

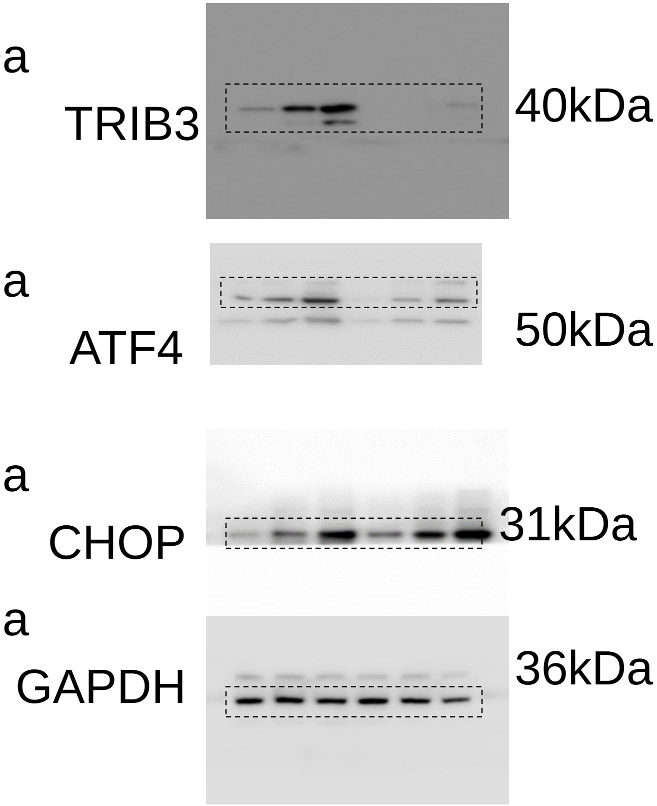

Full unedited gel for Figure 5I

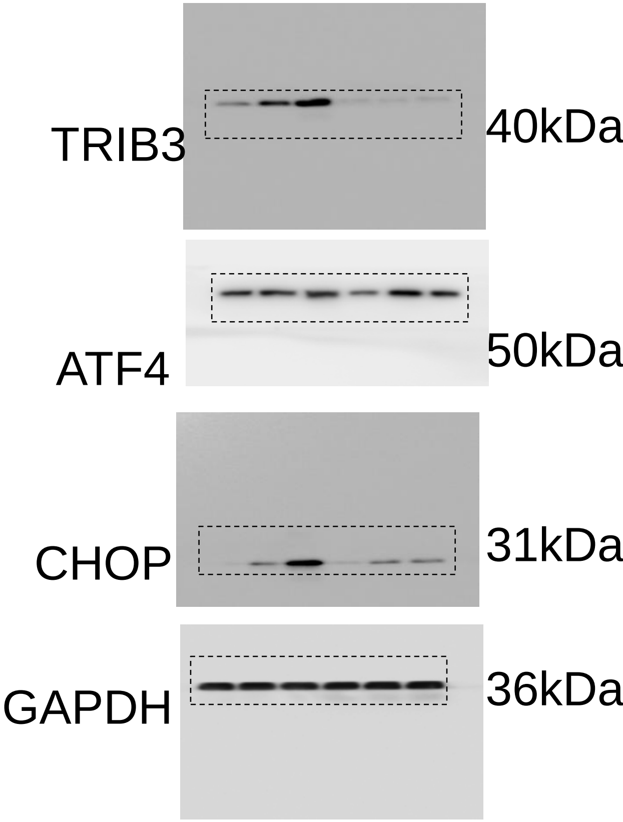

Full unedited gel for Supplementary Figure 1A

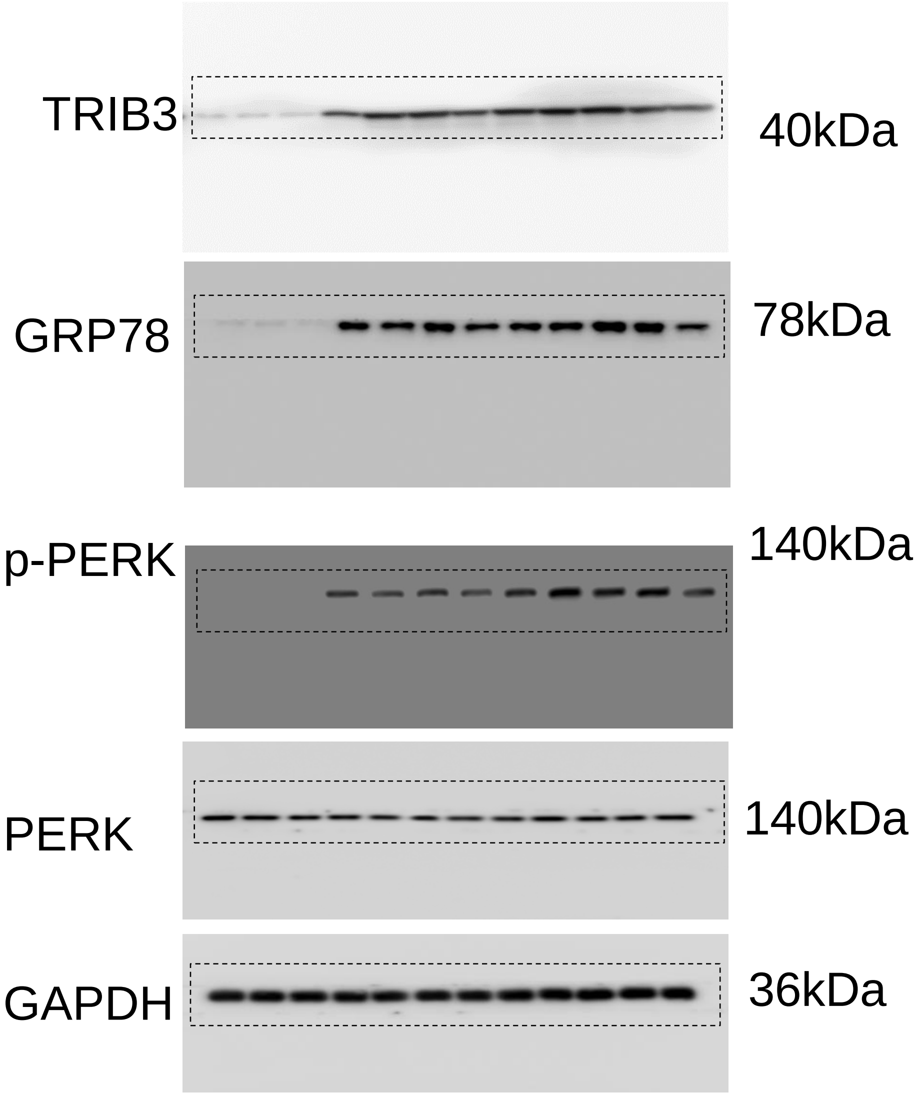

Full unedited gel for Supplementary Figure 4D

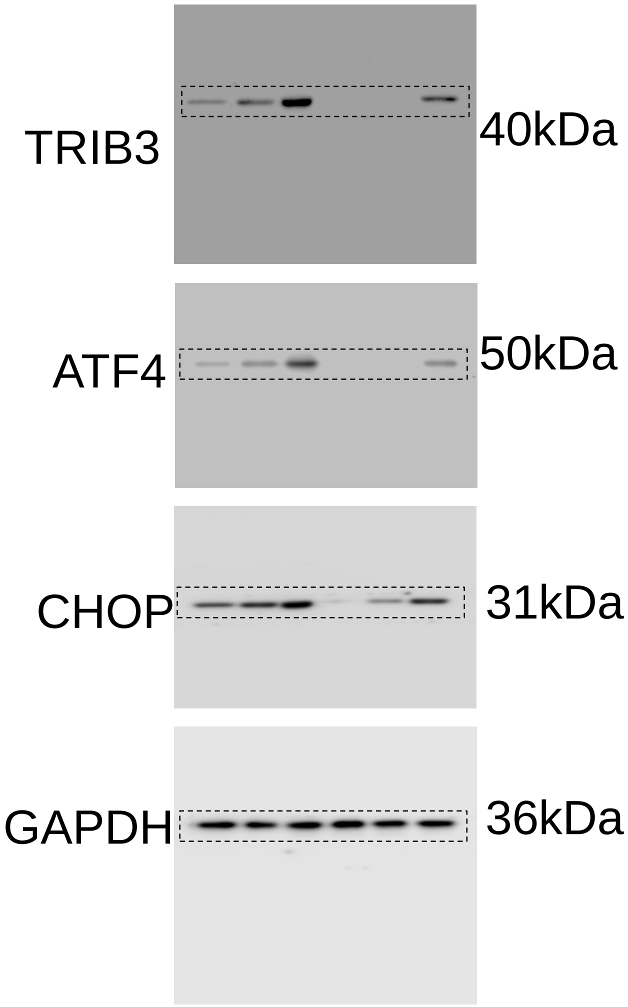

Full unedited gel for Supplementary Figure 4E

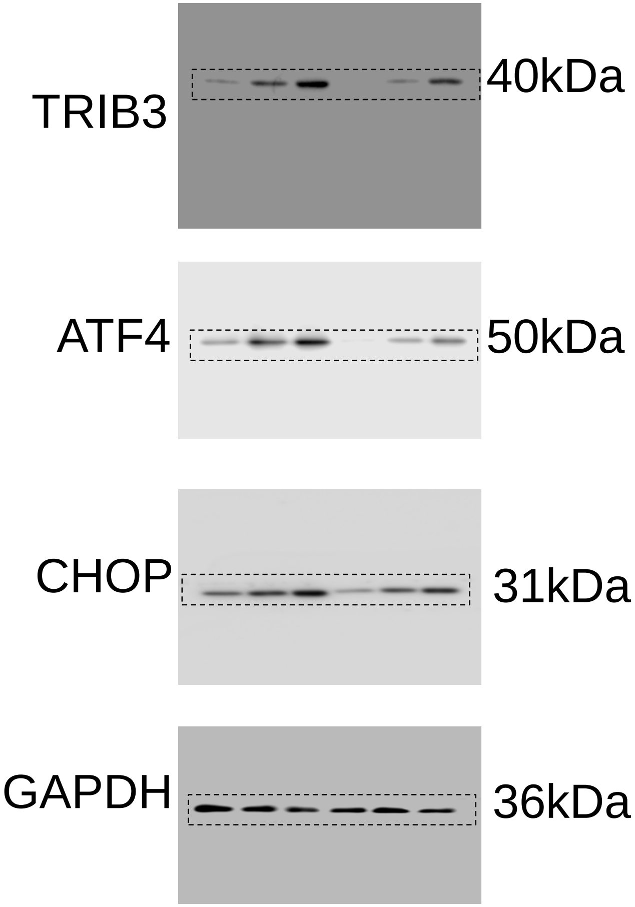

Full unedited gel for Supplementary Figure 4F

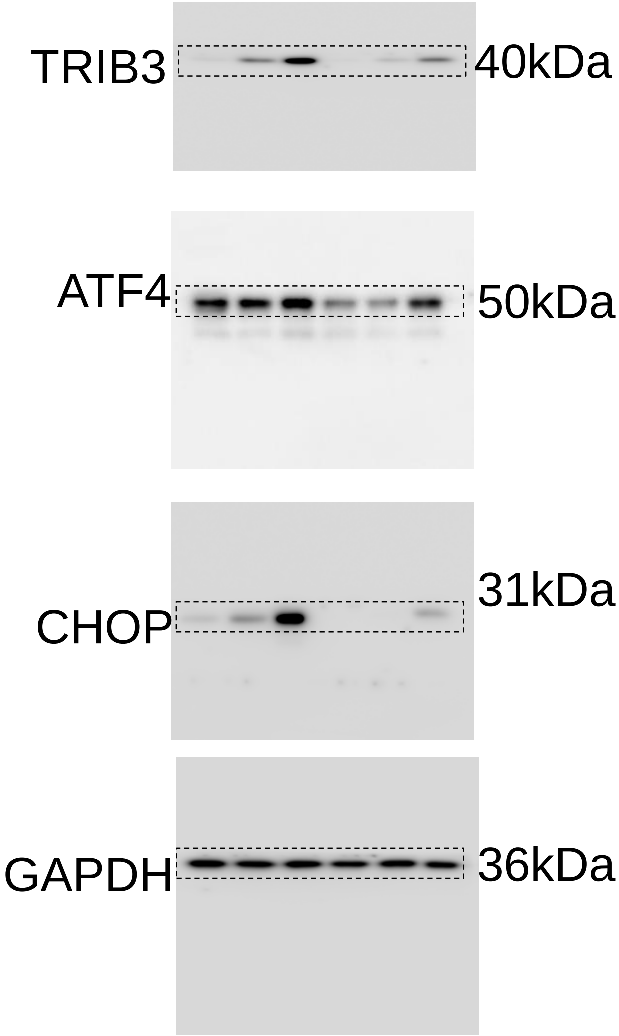

Full unedited gel for Supplementary Figure 10

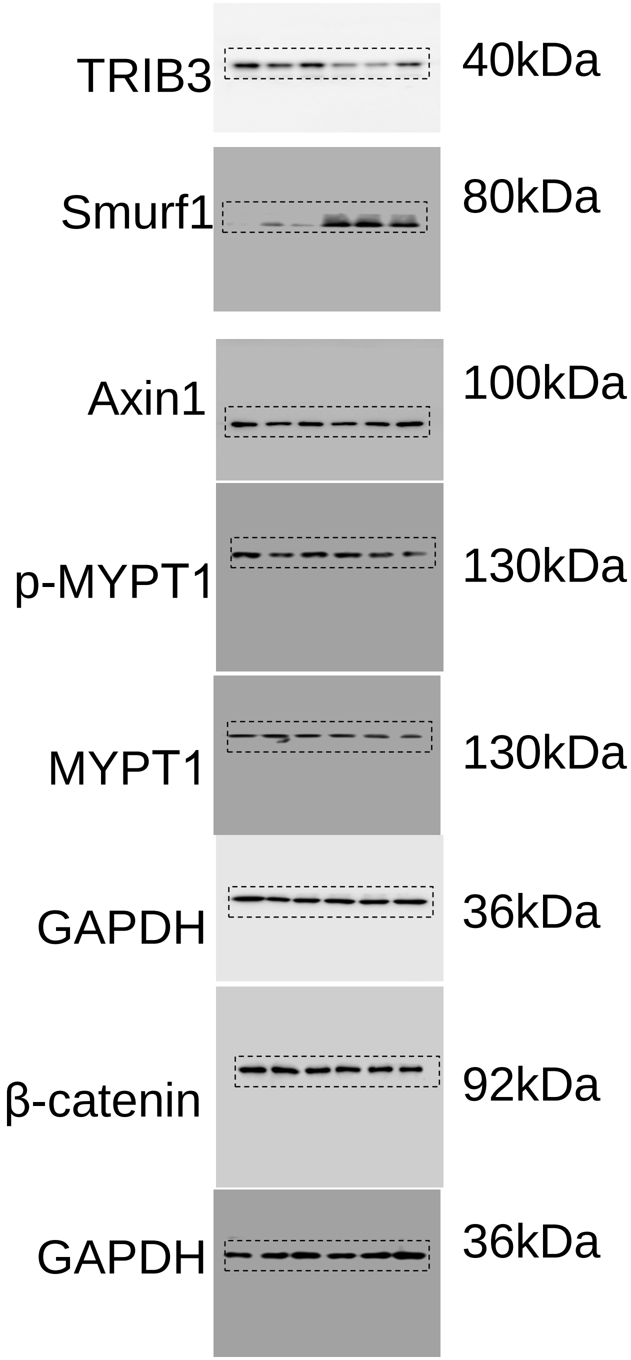

Supplement: Unedited blot and gel images [file jci-135-175972-s255.pdf]
